# Tumor Heterogeneity Underlies Differential Cisplatin Sensitivity in Mouse Models of Small-Cell Lung Cancer

**DOI:** 10.1016/j.celrep.2019.05.057

**Published:** 2019-06-11

**Authors:** Franziska Böttger, Ekaterina A. Semenova, Ji-Ying Song, Giustina Ferone, Jan van der Vliet, Miranda Cozijnsen, Rajith Bhaskaran, Lorenzo Bombardelli, Sander R. Piersma, Thang V. Pham, Connie R. Jimenez, Anton Berns

**Affiliations:** 1 Oncode Institute, Division of Molecular Genetics, The Netherlands Cancer Institute, 1066 CX Amsterdam, the Netherlands; 2 Division of Molecular Genetics, The Netherlands Cancer Institute, 1066 CX Amsterdam, the Netherlands; 3 Department of Animal Pathology, The Netherlands Cancer Institute, 1066 CX Amsterdam, the Netherlands; 4 Amsterdam UMC, Vrije Universiteit Amsterdam, Department of Medical Oncology, Cancer Center Amsterdam, 1081 HV Amsterdam, the Netherlands

**Keywords:** SCLC, mouse models, tumor heterogeneity, cisplatin, chemotherapy, RNA-seq, transcriptomics, mass spectrometry, proteomics

## Abstract

Small-cell lung cancer is the most aggressive type of lung cancer, characterized by a remarkable response to chemotherapy followed by development of resistance. Here, we describe SCLC subtypes in Mycl- and Nfib-driven GEMM that include CDH1-high peripheral primary tumor lesions and CDH1-negative, aggressive intrapulmonary metastases. Cisplatin treatment preferentially eliminates the latter, thus revealing a striking differential response. Using a combined transcriptomic and proteomic approach, we find a marked reduction in proliferation and metabolic rewiring following cisplatin treatment and present evidence for a distinctive metabolic and structural profile defining intrinsically resistant populations. This offers perspectives for effective combination therapies that might also hold promise for treating human SCLC, given the very similar response of both mouse and human SCLC to cisplatin.

## Highlights

• Heterogeneity and plasticity of SCLC underlies differential sensitivity to cisplatin
• Cisplatin selectively eradicates the CDH1-negative, NFIB-positive SCLC compartment
• Identification of intrinsically cisplatin-resistant CDH1-high alveolar lesions
• Multi-omics analysis identifies distinctive metabolic circuitry in resistant populations

## Graphical Abstract

**Figure:**
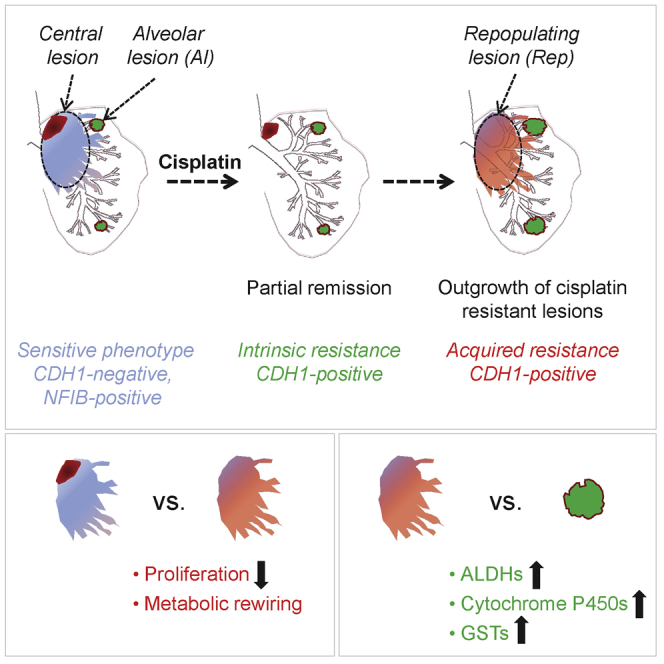

## Teaser

Böttger et al. show the presence of histologically and molecularly distinct sub-populations of small-cell lung cancer (SCLC) in advanced mouse models. They demonstrate that the heterogeneity of SCLC is responsible for the differential sensitivity to cisplatin and identify metabolic circuitries that likely contribute to cisplatin resistance.

## Introduction

Small-cell lung cancer (SCLC) is the most aggressive type of lung cancer, with dismal prognosis for patients (Gazdar et al., 2017, Semenova et al., 2015). It is characterized by early and widespread metastatic dissemination and, strikingly, a remarkable response to platinum-based chemotherapy followed almost invariably by development of resistant disease. The first-line therapy for SCLC has not changed over several decades, and there is no effective second-line therapy to date (Farago and Keane, 2018, Koinis et al., 2016, Rossi et al., 2018). Importantly, mechanisms underlying initial sensitivity and subsequent resistance of SCLC cells are not understood, and SCLC remains a recalcitrant cancer (Ujhazy and Lindwasser, 2018, Yang et al., 2015).

It has been well documented that one of the likely cells of origin of SCLC is a rare neuroendocrine (NE) cell (Gazdar et al., 1980, Park et al., 2011, Sutherland et al., 2011). NE cells are located throughout the pulmonary tree and can be found either as single cells or in clusters called NE bodies (Kumar et al., 2017, Kuo and Krasnow, 2015). Because human SCLC is often diagnosed late in the course of the disease, computed tomography (CT) scans of SCLC patients often reveal a bulky central mass with lymph node (LN) involvement, which makes the identification of primary tumor origin difficult. In addition, early metastatic spread of SCLC guides the treatment decision toward chemotherapy rather than resection, leading to sparsity of biological material for analysis (Gazdar et al., 2017). In this context, mouse models provide unique tools that allow dissection of SCLC initiation, progression, and drug response (Semenova et al., 2015).

The first mouse model of SCLC was based on conditional inactivation of key tumor suppressors *Rb1* and *Trp53* (RP mice), which are mutated in almost all SCLC tumors (George et al., 2015, Meuwissen et al., 2003). The tumors closely resemble the human disease in both their histopathology and metastatic signature (Meuwissen et al., 2003). A number of laboratories have developed additional models in which tumor development is driven by deletion of other relevant tumor suppressors, such as *Pten* and *Rbl2*, or by the expression of other oncogenes, such as *Mycl*, *cMyc*, and *Nfib* (Mollaoglu et al., 2017, Semenova et al., 2016, Wu et al., 2016).

We have recently generated and described two mouse models of SCLC, overexpressing *Mycl* and *Nfib* (Huijbers et al., 2014, Semenova et al., 2016), following our finding that *Mycl* and *Nfib* oncogenes are frequently amplified and/or overexpressed in RP mouse tumors (RPM and RPF mice, respectively). We and others also showed that these oncogenes are frequently overexpressed in human SCLC (Denny et al., 2016, George et al., 2018, Semenova et al., 2016). In line with this, forced overexpression of *Mycl* or *Nfib* gave rise to accelerated tumor development in mouse models of SCLC (Huijbers et al., 2014, Semenova et al., 2016). Importantly, overexpression of these oncogenes also altered the tumor growth pattern and tumor heterogeneity profile.

In this study we characterize tumor heterogeneity and drug response in these models and provide evidence that heterogeneity underlies cisplatin sensitivity and may explain both the remarkable initial sensitivity and subsequent resistance to chemotherapy that is frequently observed in SCLC patients.

## Results

### Tumor Heterogeneity in SCLC Mouse Models

Our laboratory has established several mouse models of SCLC, including *Rb1*/*Trp53* (RP, control, *Rb1*^*flox/flox*^*;Trp53*^*flox/flox*^), *Rb1*/*Trp53/Mycl* (RPM, *Rb1*^*flox/flox*^*;Trp53*^*flox/flox*^*;CAG < Lox66Mycl-LucLox71* >), and *Rb1*/*Trp53*/*Nfib* (RPF, *Rb1*^*flox/flox*^*;Trp53*^*flox/flox*^*;CAG < Lox66Nfib-LucLox71* >). Upon Cre-mediated recombination, *Rb1* and *Trp53* are both deleted, whereas either *Mycl* or *Nfib* is expressed along with luciferase by the CAG promoter introduced into the *ColA1* locus. As described previously, all mouse models develop SCLC with tumor cells expressing markers of NE differentiation (Huijbers et al., 2014, Meuwissen et al., 2003, Semenova et al., 2016).

Here, we set out to explore the extent of tumor diversity and heterogeneity that arises in these models when these genes are switched in a range of cell types in lung by intratracheal injection of Adeno-CMV-Cre virus.

**Figure 1:**
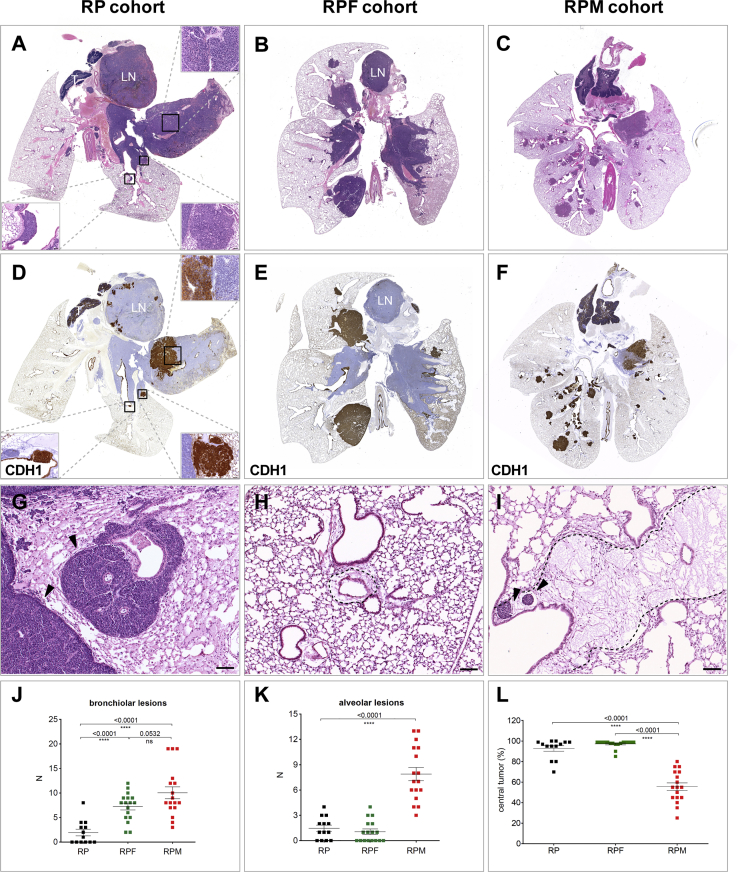
Tumor Heterogeneity in Mouse Models of SCLC (A) Representative H&E staining of RP control lung (n = 13 mice). Upper right inset shows an area of the central tumor with two tumor populations in close proximity (scale bar, 50 μm). Lower right inset shows an alveolar lesion, lower left, a bronchial lesion (scale bar, 20 μm). LN, lymph node; T, thymus. (B) Representative H&E staining of RPF lung (n = 17 mice) with large central and disseminating lesions. (C) Representative H&E staining of RPM lung (n = 17 mice), showing central and numerous peripheral lesions. (D–F) CDH1 staining of RP (D), RPF (E), and RPM (F) lungs, demonstrating the heterogeneity of positive (brown) and negative (blue) populations among the SCLCs. (G) H&E staining showing intrapulmonary metastasis lesions within peri-vascular and peri-bronchiolar space of the lung of RP animals; arrowheads indicate sheath growth pattern. (H) Peri-vascular and peri-bronchiolar space of the lung of a non-tumor-bearing animal. (I) Peri-vascular and peri-bronchiolar space of the lung of a tumor-bearing animal showing edematous change; arrowheads indicate tumor cells within lymph vessels. LN, lymph node. Scale bars for (G)–(I), 100 μm. (J and K) Quantification of bronchiolar (J) and alveolar (K) lesions. (L) Percentage of total tumor area within the lung occupied by the central tumor compartment. See also Figure S1.

In the lungs of RP animals, we frequently identified bulky tumor masses in the central hilar area, as well as a few lesions within the bronchial, bronchiolar, and alveolar space (Figure 1A). Central tumor constituted the majority (mean 93%) of the total tumor burden (Figure 1L). The tumor cells grew in a sheet-like arrangement and frequently invaded mediastinal LNs (Figure 1A). Further analysis of the central compartment revealed frequent juxtaposition of what appeared to be two distinct tumor populations readily distinguishable by the expression of E-cadherin (CDH1) (Figures 1A and 1D). CDH1^hi^ cells were mostly NFIB^lo^ CGRP^hi^, while CDH1^neg^ cells were invariably NFIB^hi^ CGRP^lo^ (Figures S1A and S1B). All tumors, irrespective of genotype, initially expressed high levels of CDH1 (Figure S1C), and only later, in the course of tumor development, did CDH1^neg^ populations appear. We therefore consider CDH1^hi^ lesions to be primary, giving rise to CDH1^neg^ intrapulmonary metastasis. Of note, lesions found in the bronchial and bronchiolar tree and within the alveolar space were always CDH1^hi^ and also represented primary tumors (Figure 1D and data not shown).

The tumor cells constituting intrapulmonary metastasis showed classical small-cell morphology, with coarse nuclear chromatin, minimal cytoplasm, and high mitosis (Figures 1A and 1G). They filled lymph vessels, identified by podoplanin (PDPN) staining (Figures S1D and S1E), and grew in a sheath arrangement within peri-vascular and peri-bronchiolar areas, disseminating within intra- as well as inter-lobular spaces (Figure 1G and data not shown). Noticeably, compared with a healthy lung, peri-vascular/peri-bronchial areas in the lungs of SCLC tumor-bearing mice often showed marked edematous changes (Figures 1H and 1I).

In the RPF model, as in RP, the main tumor burden was represented by a central compartment with a mean of 97% (Figure 1L). At the same time, we observed a shift toward CDH1^neg^/NFIB^hi^ intrapulmonary metastasis in the centrally located tumors, showing more abundant sheath structures (Figure 1E and data not shown). Interestingly, quantification of bronchiolar lesions showed a clear increase, with an average of seven in RPF mice compared with two in RP control lesions per lung section (Figure 1J). In contrast, the number of alveolar lesions was similar to that of control (Figure 1K).

**Figure 2:**
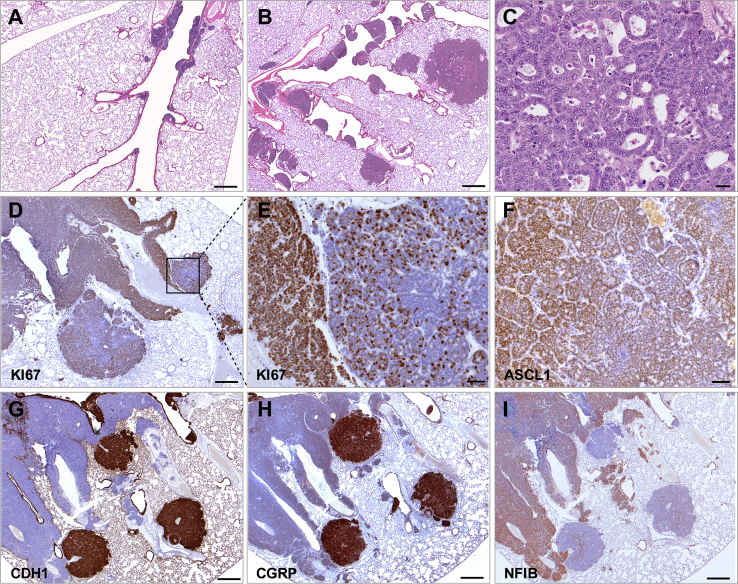
MYCL Promotes Development of NE Lesions in the Alveolar Space (A) Part of a bronchial tree in an RP mouse (n = 13 mice) showing several bronchial lesions. (B) Part of a bronchial tree in an RPM mouse (n = 17 mice) showing multiple bronchial and alveolar lesions. (C) Alveolar lesion showing pseudo-glandular structures. (D) Ki67 staining showing proliferating cells within alveolar lesion and within the adjacent intrapulmonary metastasis lesion. (E) Magnification of the area within (D). (F) ASCL1 staining of alveolar lesion. (G–I) CDH1 (G), CGRP (H), and NFIB (I) staining of three alveolar lesions in close proximity to intrapulmonary metastasis lesions (sequential sections). Scale bar for (A), (B), (D), and (G)–(I), 500 μm. Scale bar for (C), 20 μm. Scale bar for (E) and (F), 50 μm. See also Figure S1.

The central tumor compartment in RPM animals was qualitatively similar to that in the RP control, often displaying both CDH1^hi^ and CDH1^neg^ populations (Figures 1C and 1F). However, in contrast to both RP and RPF mice, it represented on average only 56% of the total tumor burden (Figure 1L). Instead, in addition to central lesions, RPM lungs presented with numerous independently arising primary lesions found at all levels of the bronchial and bronchiolar tree and within the alveolar space (Figures 1C and 1F). The most prominent increase (7 times compared with both RP and RPF mice) was observed in the number of alveolar lesions (Figures 1K, 2A, and 2B). The differential effects of *Nfib* and *Mycl* on the bronchiolar and alveolar lesions suggest that the cells from which they originate are different, thereby further expanding the number of cells of origin of SCLC.

Alveolar space lesions consisted of cells with relatively rich cytoplasm and fine nuclear chromatin and often formed organized nests and/or pseudo-glandular structures (Figure 2C). They displayed reduced proliferation compared with intrapulmonary metastasis and were less invasive, showing clear lesion demarcations (Figures 2D and 2E). They were ASCL1 positive and predominantly CDH1^hi^/CGRP^hi^/NFIB^lo^ (Figures 2F–2I and S1F).

Thus, we identified substantial heterogeneity in all mouse models of SCLC, with NFIB promoting progression of central primary lesions to disseminated intrapulmonary metastasis and causing an increase in the number of bronchiolar lesions and with MYCL stimulating tumor initiation and progression at multiple locations along the pulmonary tree. Interestingly, only *Mycl* overexpression gave rise to a large increase in the number of alveolar lesions. Noticeably, these lesions had rather consistently high ASCL1 but low NEUROD1 expression (Figure S2B).

### Differential Sensitivity of Tumor Population to Cisplatin Treatment

**Figure 3:**
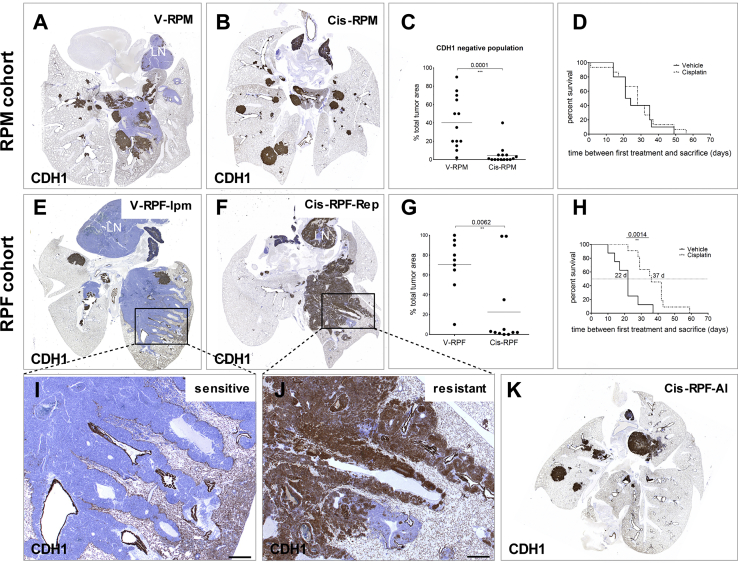
Differential Sensitivity of Distinct Tumor Populations to Cisplatin Treatment (A) Representative CDH1 staining of RPM lung treated with vehicle (n = 13 mice). (B) Representative CDH1 staining of RPM lung treated with cisplatin (n = 15 mice). (C) Quantification of the area of the lung occupied by CDH1-negative tumor. (D) Survival curves for vehicle- and cisplatin-treated RPM animals; time 0 is set at the day of the first treatment. (E) Representative CDH1 staining of RPF lung treated with vehicle (n = 9 mice). (F) Representative CDH1 staining of RPF lung treated with cisplatin (n = 11 mice). (G) Quantification of the area of the lung occupied by CDH1-negative tumor. (H) Survival curves for vehicle- and cisplatin-treated RPF animals; time 0 is set at the day of the first treatment. (I and J) Magnification of (I) an intrapulmonary metastasis (Ipm; vehicle-treated animal) and (J) a repopulating (Rep; cisplatin-treated animal) tumor. (K) Image of cisplatin-treated RPF lung showing predominantly alveolar lesions (Al) after treatment. Scale bar for (I) and (J), 500 μm. See also Figure S2 and Table S1.

To test the sensitivity of these tumor populations to chemotherapy, we treated RPM mice with cisplatin, a prominent component of first-line therapy for SCLC patients. We chose the RPM model because it presented with the broadest tumor heterogeneity. In two independent experiments, a total of 15 animals were treated with cisplatin (Cis-RPM) and 13 with vehicle (V-RPM). We used bioluminescence imaging (which becomes co-expressed upon *Mycl* transgene activation) to decide on the start of treatment: when the bioluminescence signal reached 1 × 10^5^ relative luciferase units, a level that corresponds to a significant tumor burden (data not shown), cisplatin was given intravenously once every 2 weeks at a dose of 6 mg/kg. Treatment was continued until animals developed breathing problems, which was on average after 3 cycles; Table S1). We next analyzed the lungs of vehicle- and cisplatin-treated animals with a panel of lineage and/or differentiation markers (Figure S2A), which did not show any substantial difference upon cisplatin treatment. However, this analysis revealed a significant reduction, and in several cases a complete absence, of the CDH1^neg^/NFIB^hi^/CGRP^lo^ intrapulmonary metastasis population (Figures 3A and 3B). In contrast, the CDH1^hi^ central compartment and, most strikingly, the peripheral bronchial and alveolar lesions seemed unaffected (Figures 3A and 3B). We quantified this by calculating the relative pulmonary tumor burden represented by the CDH1^neg^ population (Figure 3C). Despite these changes, treatment of RPM mice did not result in a significant survival benefit (Figure 3D).

### Treatment of NFIB-Expressing RPF Mice Results in Significant Survival Benefit and Development of Resistant Tumor with Altered Phenotype

We reported previously that the majority of high-grade human NE tumors expressed high levels of NFIB, highlighting the relevance of the RPF mouse model for understanding SCLC behavior in patients (Semenova et al., 2016). As mentioned earlier, RPF mice show a prominent expansion of the CDH1^neg^/NFIB^hi^/CGRP^lo^ tumor population (Semenova et al., 2016). Therefore, in view of the results obtained following the treatment of RPM cohort, we anticipated a better response to treatment of RPF animals, as they present with the “sensitive” tumor phenotype. To test this, we treated RPF animals following a schedule identical to that of RPM treatment. Eleven RPF animals were treated with cisplatin and 9 with vehicle. As hypothesized, and in contrast to the RPM cohort, cisplatin treatment conveyed a significant survival benefit (Figure 3H), suggesting that NFIB-expressing tumors responded better to treatment. Histopathological evaluation of the tumors in both vehicle- and cisplatin-treated groups showed that vehicle-treated RPF (V-RPF) mice presented with mostly CDH1^neg^ or CDH1^lo^ tumors (Figures 3E and 3G). Interestingly, as was the case for RPM animals, the majority of tumors in cisplatin-treated lungs were CDH1^hi^ (Figures 3F, 3G, and 3K). Moreover, we observed two tumor phenotypes in the cisplatin-treated cohort. One phenotype was represented by CDH1^hi^ tumor that displayed an intrapulmonary metastasis-like growth pattern (termed repopulating lesion [RPF-Rep]), a tumor population that in all vehicle-treated cases was invariably CDH1^neg^ (Figure 3F). The second phenotype corresponded to peripheral nodular bronchiolar and alveolar CDH1^hi^ lesions (RPF-Al) (Figure 3K).

### Transcriptomic and Proteomic Analyses of NFIB-Expressing Tumors

We next performed in-depth gene expression analyses of vehicle- and cisplatin-treated tumor populations that arose in RPF mice. Two separate comparative analyses were carried out. In the first, vehicle-treated central lesions (V-RPF) were compared with cisplatin-treated RPF-Rep lesions (Cis-RPF-Rep) that had repopulated the corresponding regions in V-RPF mice, with the aim of unraveling biological processes associated with cisplatin resistance. In the second, differences between the two distinct cisplatin-resistant tumor populations, Cis-RPF-Rep and Cis-RPF-Al, were compared. Both RNA and proteins were extracted from defined areas of paraffin-embedded, immunohistochemically characterized lung tumor samples of five vehicle-treated and seven cisplatin-treated (four Cis-RPF-Rep and three Cis-RPF-Al) mice (Figure S3A). RNA sequencing (RNA-seq) identified 15,462 genes in total, 5,143 of which were also identified at the protein level using tandem mass spectrometry (5,686 unique proteins were identified in total) (Figure S3B). Unsupervised hierarchical cluster analysis using normalized spectral counts for identified proteins demonstrated that each of the three tumor populations (V-RPF, Cis-RPF-Rep, and Cis-RPF-Al) was characterized by a distinct proteome profile. With the exception of three outlier samples likely due to experimental fluctuations in protein input as judged by gel images (Figure S3C), all samples clustered according to experimental condition: cisplatin-treated clustered away from vehicle-treated samples, and within the treated cohort, RPF-Al clustered separately from RPF-Rep tumor samples (Figure S3D). At the RNA level, unsupervised hierarchical cluster analysis using normalized read counts separated vehicle-treated from cisplatin-treated populations, but there was no further separation between RPF-Rep and RPF-Al within this treated sample group (Figure S3E).

### Cisplatin-Treated Samples Display Reduced Proliferation and a Metabolic Switch

**Figure 4:**
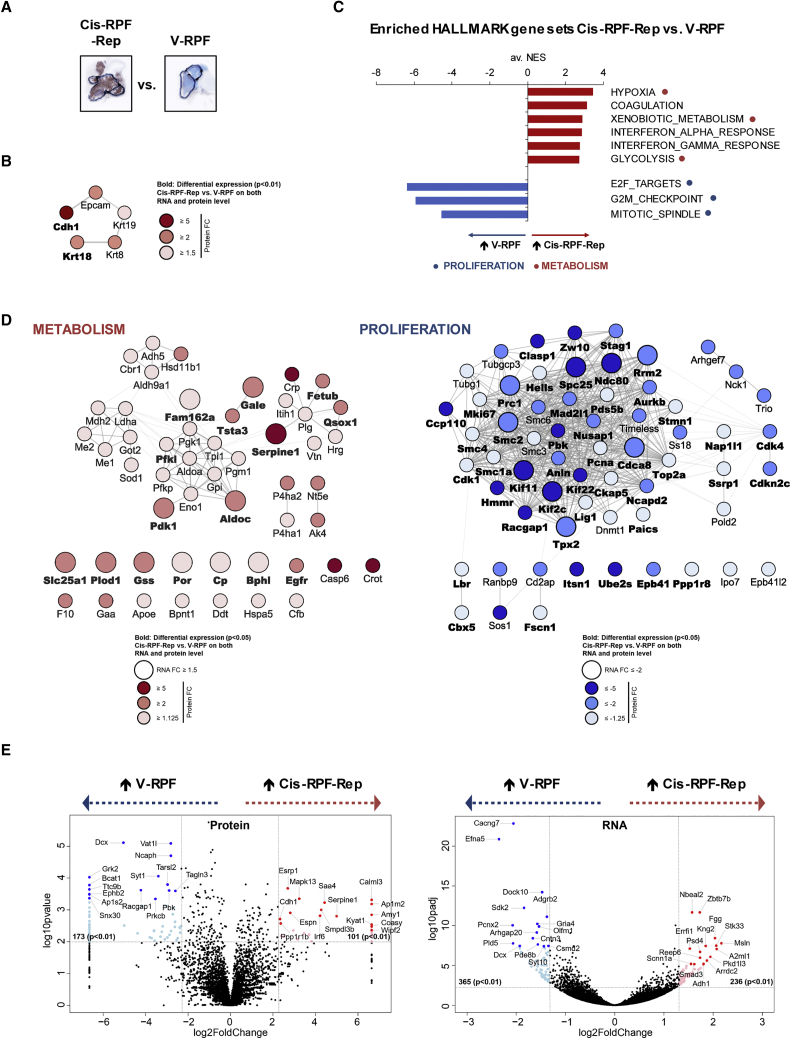
Cisplatin-Treated RPF Samples Display Reduced Proliferation and a Metabolic Switch (A) Representative images (CDH1 staining) of samples used for Cis-RPF-Rep versus V-RPF comparison (for images of all five V-RPF and four Cis-RPF-Rep mice analyzed, see Figure S3A). (B) Functional interaction cluster of significantly (p < 0.01) differentially expressed proteins (determined using beta-binomial test on normalized spectral counts) Cis-RPF-Rep versus V-RPF associated with *Cdh1* (STRING database). Node color corresponds to fold change (FC) at the protein level and edge thickness to confidence of STRING interaction (thinnest = high confidence [≥0.7], thickest = highest confidence [≥0.9]). Gene names in boldface type (*Cdh1* and *Krt18*) correspond to proteins that are significantly (p < 0.01) differentially expressed on both protein and RNA level (for RNA-seq data, DESeq2 was used for normalization and differential expression analysis). (C) Common gene sets significantly (FDR q value < 0.05, normalized enrichment score [NES] ≥ 2.5) enriched at both the protein and RNA levels in Cis-RPF-Rep versus V-RPF comparison (GSEA_HALLMARKS). NES values of protein and RNA analyses were averaged for representative purposes (av. NES); separate protein and RNA enrichment graphs can be found in Figure S5B. (D) Network analysis of differentially expressed genes (DEGs; unadjusted p < 0.05), on the basis of proteins contributing to core enrichment of gene sets HYPOXIA, XENOBIOTIC_METABOLISM, and GLYCOLYSIS (“metabolism”) and E2F_TARGETS, G2M_CHECKPOINT, and MITOTIC_SPINDLE (“proliferation”), respectively. Marked in boldface type are genes that were significantly differentially expressed at both the RNA and protein levels. Node color reflects FC at the protein level and node size FC at the RNA level. (E) Most discriminatory genes in Cis-RPF-Rep versus V-RPF comparison. All genes with p values < 0.01 and −5 ≥ FC ≥ 5 (protein, left plot) or −2.5 ≥ FC ≥ 2.5 (RNA, right plot) are colored in light blue (UP in vehicle-treated) or light red (UP in cisplatin-treated RPF-Rep), respectively. Highlighted with proteins names (and marked with dark blue and red dots) are the 15 most highly differentially expressed genes in each direction (by p value after FC filtering). See also Figures S3–S5 and Tables S2, S3, and S4.

In a search for gene expression profiles associated with cisplatin resistance, we first compared vehicle-treated samples (V-RPF) with cisplatin-treated repopulating lesions (RPF-Rep) (Figure 4A). Differentially expressed genes (DEGs) with p values < 0.01 were selected. In total, 274 and 601 DEGs were identified at the protein and RNA levels, respectively (Figure S4A). Of 274 differentially expressed proteins, 101 were more abundant in the cisplatin-treated RPF-Rep population. Among these, *Cdh1* was 1 of the 10 genes with increased abundance at both the RNA and protein levels (Figure S4A). With a 5-fold increase in abundance following cisplatin treatment, *Cdh1* was also among the top 15 most strongly enriched proteins (Figure 4E), in line with the differential staining pattern observed in immunohistochemical analysis. Intriguingly, along with *Cdh1* (fold change [FC] 5.0), epithelial marker proteins *Epcam* (2.7), *Krt8* (2.5), *Krt18* (2.5), and *Krt19* (1.8) were significantly enriched at the protein level and formed a high-confidence functional interaction cluster (STRING interaction score ≥ 0.7) (Figures 4B and S4B). Immunohistochemical analysis using an anti-wide-spectrum keratin (KWS) antibody further supported the observation of a shift toward an epithelial phenotype in the cisplatin-treated lung cancer population (Figure S4C). To gain further functional insight into the cisplatin resistance profile, an unbiased gene set enrichment approach was used. Following ranking of all identified genes on the basis of a combined p value and FC score, significantly enriched HALLMARK gene sets were identified (false discovery rate [FDR] q value < 0.05, normalized enrichment score [NES] ≥ 2.5). Besides an increase in coagulation and interferon response, likely a direct consequence of cisplatin treatment, the most striking change observed at both the RNA and protein levels was a metabolic switch, as highlighted by an enrichment in gene sets HYPOXIA, XENOBIOTIC_METABOLISM, and GLYCOLYSIS (Figures 4C, S5A, and S5B), and represented by DEGs such as *Pdk1*, *Aldoc*, *Slc25a1*, and *Gss* (p < 0.05) (Figure 4D, left panel). Altered expression of key metabolic components and, concomitant with this, a switch in cellular energy homeostasis have been suggested as a common mechanism for response and adaptation to cisplatin therapy in various cancer types (Morandi and Indraccolo, 2017). In this context, the recent finding that *Slc25a1* plays a key role in the drug-induced metabolic switch that enables tumor cells to become resistant to cisplatin (Fernandez et al., 2018) is of particular interest and in agreement with our observations. Furthermore, it has been shown that cell-cell contacts mediated by proteins such as CDH1 can upregulate PI3K/AKT signaling (De Santis et al., 2009), and activation of the PI3K survival pathway is associated with both glycolysis and platinum resistance. Staining for p-AKT showed variable and sometimes intense regional staining, with a tendency to be higher in cisplatin-treated samples (Figure S2A). RNA-seq analysis showed a more convincing difference, with AKT-activated oncogenic gene signatures AKT_UP_MTOR_DN.V1_UP and AKT_UP.V1_UP significantly enriched (Figure S5C).

Inversely, 173 of the 274 differentially expressed proteins were significantly less abundant in the cisplatin-treated RPF-Rep populations. Proliferation marker *Mki67* was among the 62 genes with decreased abundance at both the RNA and protein levels following cisplatin treatment (Figures S4A). More than a quarter of these 62 genes (26%) were cell cycle- and proliferation-associated genes. Accordingly, E2F-TARGETS, G2M_CHECKPOINT, and MITOTIC_SPINDLE were the three most negatively enriched gene sets (Figures 4C and 4D, right panel; Figures S5A and S5B), in line with cisplatin’s inherent mode of action as a DNA-binding and proliferation-impairing agent. In further support, levels of mitotic marker phospho-histone H3 (p-HH3) were reduced in the RPF-Rep population in immunohistochemical analysis (Figure S4C).

In addition to the marked reduction in abundance of many proliferation-associated genes, another noteworthy observation was that the 2 genes with the strongest reductions in abundance in the cisplatin-treated population, doublecortin/*Dcx* at the protein and ephrin-A5/*Efna5* at the RNA level (Figure 4E), are both neuronal lineage markers. In addition to being the most differentially expressed protein, *Dcx* was also among the top 15 genes most strongly reduced at the RNA expression level (Figure 4E, right panel). Moreover, a wide set of neuronal differentiation and migration-associated genes such as *Ncam1*, *Zeb1* (both RNA and protein level), drebrin/*Dbn1*, *Ephb2*, *Rufy3* (protein), netrin-1 receptor/*Dcc*, neuroligin-1/*Nlgn1*, *Robo1*, contactins *Cntn3* and *Cntn4*, and semaphorins *Sema5b* and *Sema6d* (RNA) were significantly less abundant in the cisplatin-treated lung tumors (Figures S4D and S4E), suggesting that cisplatin causes tumors to shift toward less pronounced neuronal phenotype.

### Comparison of the Two Cisplatin-Treated Populations Highlights a Unique Alveolar Lesion Identity

**Figure 5:**
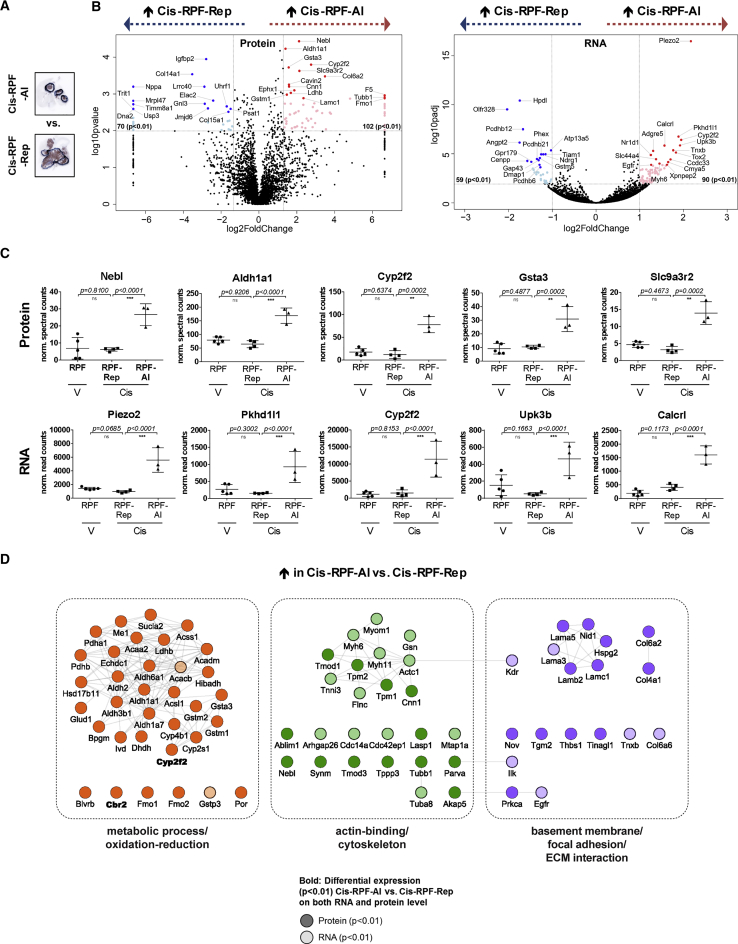
Comparison of the Two Cisplatin-Treated RPF Populations Highlights a Unique Alveolar Lesion Identity (A) Representative images (CDH1 staining) of samples used for RPF-Al versus RPF-Rep comparison (for images of all three RPF-Al and four RPF-Rep mice analyzed, see Figure S3A). (B) Most discriminatory genes in RPF-Al versus RPF-Rep comparison (determined using beta-binomial test for protein and DESeq2 for RNA-seq data). All genes with p values < 0.01 and −2.5 ≥ fold change (FC) ≥ 2.5 (protein, left plot) or −2 ≥ FC ≥ 2 (RNA, right plot) are colored in light blue (UP in RPF-Rep) or light red (UP in RPF-Al), respectively. Highlighted with proteins names (and marked with dark blue and red dots) are the 15 most highly differentially expressed genes in each direction (by p value after FC filtering). (C) Expression plots of the 5 most differential genes on protein (top row) and RNA level (bottom row), respectively (^∗^p < 0.01, ^∗∗^p < 0.001, and ^∗∗∗^p < 0.0001; n.s., not significant). (D) Highly differentially expressed proteins (p < 0.01 at the protein and/or RNA level) form three highly interactive functional clusters: metabolic process and oxidation-reduction (left), actin-binding and cytoskeleton (middle), and basement membrane, focal adhesion, and ECM interaction (right). See also Figure S6 and Tables S2 and S3.

As mentioned earlier, we observed two distinct “resistant phenotypes” in cisplatin-treated RPF lungs, RPF-Rep and RPF-Al. We therefore set out to investigate what distinguishes these two tumor populations at the molecular level (Figures 5A and S6A). The substantial number of highly DEGs (p < 0.01) between RPF-Al and RPF-Rep on protein and RNA level (172 and 149, respectively), as well as the unsupervised separation of these two populations on the basis of protein expression (Figure S3D), indicate that they are distinct subtypes. Of 172 significantly differentially expressed proteins, 102 were more abundant in the RPF-Al population. Strikingly, stem cell marker *Aldh1a1* was one of the most discriminatory proteins (Figures 5B, left, and Figure 5C, top panel), and part of a highly connected cluster of metabolic proteins (Figure 5D). Immunohistochemical analysis using anti-ALDH1A1 antibody confirmed that it was indeed expressed more abundantly in RPF-Al compared with RPF-Rep (Figure S6B). Interestingly, ALDH1A1 was also strongly expressed by alveolar lesions found in RPM lungs. Alveolar lesions from both RPM vehicle- and cisplatin-treated lungs showed equally high levels of ALDH1A1 (Figure S6B).

Metabolic proteins not only included additional members of the aldehyde dehydrogenase family (*Aldh1a7*, *Aldh3b1*, *Ald6a1*, and *Aldh2*) but also key metabolic enzymes such as *Pdhb*, *Ldhb*, and several cytochrome P450 (*Cyp2f2*, *Cyp2s1*, and *Cyp4b1*) and glutathione S-transferase (*Gsta3*, *Gstm1*, *Gstm2*, and *Gstp3* at the RNA level) family members. Of note, together with *Aldh1a1* (FC 2.6), *Cyp2f2* (FC 6.7) and *Gsta3* (FC 3.0) were among the five most highly differentially expressed proteins (Figure 5C, top panel), with *Cyp2f2* additionally being among the top five genes with the most significant increases in RNA level in RPF-Al compared with RPF-Rep (Figure 5C, bottom panel). This suggests a radically different metabolic wiring of alveolar compared with repopulating lesions and could explain the apparent intrinsic resistance of alveolar lesions to cisplatin, as many of these differentially expressed proteins are enzymes involved in drug metabolism. Targeting these highly expressed metabolic enzymes may be a suitable strategy to overcome cisplatin resistance, as has previously been demonstrated for *Aldh1a1* in non-small-cell lung cancer (NSCLC) (MacDonagh et al., 2017).

Besides this marked elevation in metabolic gene expression in RPF-Al, we found profound differences in the expression of structural components, specifically those involved in actin-binding and cytoskeleton, as well as basement membrane, focal adhesion, and ECM interaction (Figure 5D). Both core structure components of basement membranes, such as type IV collagen (*Col4a1*) and laminin (*Lama3*, *Lama5*, *Lamb1*, and *Lamc1*) as well as bridging adaptor proteins, such as perlecan (*Hspg2*) and nidogen (*Nid1*), were highly differentially expressed (p < 0.01) and increased in RPF-Al versus RPF-Rep. This is in line with the morphological differences seen on microscopic images, with alveolar lesions showing more compact, organized nests with pseudo-glandular structures, compared with the spatially and morphologically distinct sheath-like organization of the repopulating lesions. Furthermore, it is interesting to note that several members of the protocadherin family, specifically members of the protocadherin-beta gene cluster (*Pcdhb*), were significantly less abundant in RPF-Al compared with RPF-Rep (Figure 5B, right panel; Figure S6C). *Pcdhb12*, *Pcdhb21*, and *Pcdhb6* were among the top 15 genes with the most significant decreases in RNA levels in RPF-Al compared with RPF-Rep (Figure 5B, right), with 2 additional members (*Pcdhb20* and *Pcdhb13*) also being significantly decreased at p < 0.01, and a further 4 (*Pcdhb15*, *Pcdhb5*, *Pcdhb8*, and *Pcdhb11*) at p < 0.05 (Figure S6C). These cell adhesion molecules, which constitute a subfamily of non-classic cadherins, are thought to play a role in the establishment and function of specific neuronal cell-cell connections. In contrast to the neuronal differentiation and migration-associated genes that are less abundant in both cisplatin-treated RPF-Rep and RPF-Al compared with V-RPF samples (e.g., *Dcx*, *Ncam1*; Figures S4D and S4E), the expression levels of the *Pcdhb* gene cluster appear to be specifically lower in the RPF-Al sample cohort (Figure S6C).

Taken together, our proteo-transcriptomic data show that cisplatin-treated RPF mouse lung tumors display reduced proliferation and altered metabolism and highlight a shift from a neuronal to an epithelial phenotype. On the basis of unique metabolic and structural properties, the RPF-Rep and RPF-Al tumors have distinct identities reflecting different cells of origin.

### SCLC Heterogeneity in Mouse Models as Underlying Mechanism of Differential Sensitivity to Cisplatin

**Figure 6:**
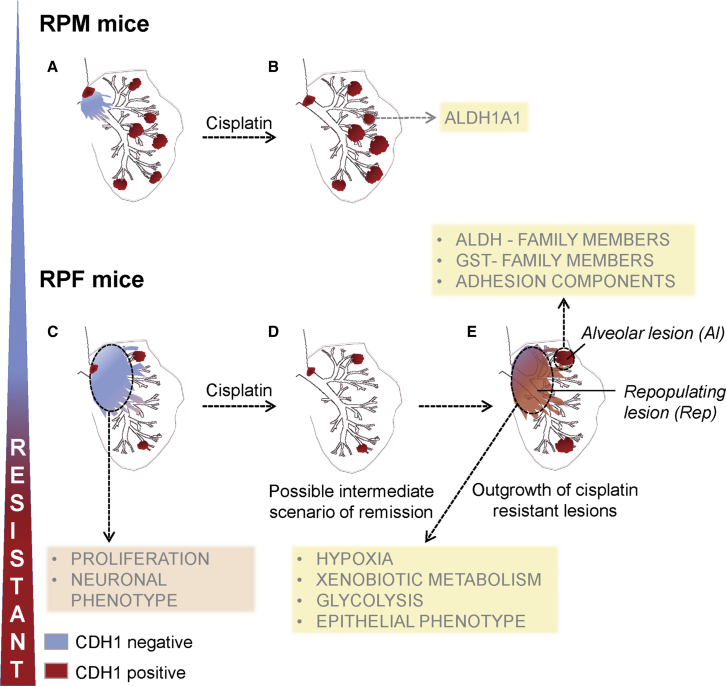
Schematic Representation of SCLC Heterogeneity in Mouse Models as Underlying Mechanism of Differential Sensitivity to Cisplatin Our results suggest that the outcome of cisplatin treatment might depend on the ratio between CDH1-positive resistant primary (brown) and CDH1-negative intrapulmonary metastasis (blue) compartments within SCLC lesions. (A and B) In RPM mice, in which CDH1-positive compartment is on average predominant (A), the regression of CDH1-negative population following cisplatin treatment (B) is not sufficient to guarantee a longer survival. (C and D) In RPF mice, the sensitive CDH1-negative central compartment (C) responds to cisplatin, and consequently its regression (D) is associated with a significant survival advantage. (E) This positive response to cisplatin is followed by subsequent growth of resistant populations, similar to what is seen in patients. Resistant tumor may represent peripheral lesions, frequently present in RPM mice, and/or central lesions that repopulate the empty peri-vascular/peri-bronchial space left behind following elimination of the sensitive population. The targeting of metabolic pathways (yellow rectangles) active in the resistant populations, in combination with cisplatin treatment, may offer an opportunity to eradicate recalcitrant SCLC.

Our combined immunohistochemical and gene expression data analyses point to tumor heterogeneity in SCLC as an important underlying mechanism of differential sensitivity to cisplatin treatment. Centrally located early lesions, initially expressing high levels of CDH1 and refractory to cisplatin, progress to highly proliferative CDH1-negative, NFIB-high lesions that are cisplatin sensitive. The regions with cisplatin responsive tumor cells are then replaced by CDH1-positive (Cis-RPF-Rep) tumors that are (or have become) refractory to cisplatin. In contrast, the peripheral bronchiolar and alveolar lesions show intrinsic cisplatin resistance (Figure 6). In RPM mice, the cisplatin-sensitive compartment constitutes only a small fraction of the tumor mass (Figure 6A), and therefore cisplatin treatment causes only partial regression (Figure 6B). Importantly, in line with the frequent *Nfib* activation in human SCLC and on the basis of our observations, the cisplatin-sensitive tumor compartment at presentation is likely the predominant one in RPF animals (Figure 6C). When exposed to chemotherapy, the sensitive NFIB-positive compartment may be effectively eliminated (Figure 6D), reflecting the initially good response, but leaving behind a “minor” resistant primary tumor compartment. Ongoing chemotherapy regimen may prevent re-establishment of the sensitive tumor but will not limit the proliferation of resistant tumor cells, or expansion of the intrinsically resistant population, explaining lack of chemotherapy response at later stages (Figure 6E).

## Discussion

SCLC is a recalcitrant disease with an urgent need for more effective treatment approaches. To date, the intrinsic complexity of SCLC as well as a lack of tumor material has limited our ability to develop effective treatment modalities. Analysis of available patient material, patient-derived xenografts (PDXs), circulating tumor cells-derived explants (CDXs), and genetically engineered mouse models (GEMMs) provides growing evidence for extensive intrinsic and acquired heterogeneity in SCLC (Borromeo et al., 2016, Calbo et al., 2011, Drapkin et al., 2018, Gazdar et al., 2015, George et al., 2015, Huang et al., 2018, Lim et al., 2017, Pozo et al., 2018, Shue et al., 2018, Zhang et al., 2018). This is further complicated by evidence for trans-differentiation and existence of combined SCLC-NSCLC (Niederst et al., 2015, Sequist et al., 2011, Zhao et al., 2018).

SCLC is characterized by a relatively good response to first-line therapy. Unfortunately, after an initial response, the majority of patients soon relapse with resistant disease. Despite concerted efforts of the international scientific and clinical community, no unequivocal underlying resistance mechanism has been found. However, some more effective drug combinations to treat SCLC have recently been described, such as the combination of standard chemotherapy together with inhibitors affecting replication stress response genes such as CHK1 and ATR (Doerr et al., 2017, Sen et al., 2017), that showed enhanced synergy in SCLC overexpressing MYC (Nagel et al., 2019). In addition, chemotherapy resistance mechanisms associated with the frequently observed loss of Schlafen11 (SLFN11) expression could be restored by EZH2 inhibition (Gardner et al., 2017), although this was not observed in a set of SCLC PDX models (Drapkin et al., 2018). However, it might require a functional immune system to benefit from SLFN11 expression (Mezzadra et al., 2019). Promising progress is being made with immunotherapy strategies showing a durable response in a small subset of patients (Hellmann et al., 2018). Our data generated in animal models support the notion that SCLC patients present with heterogeneous tumors, which at the time of diagnosis already consist of chemotherapy-sensitive and resistant tumor sub-populations. The CDH1-negative, cisplatin-sensitive population may be completely eliminated by cisplatin treatment, followed by subsequent expansion of pre-existing resistant cells. Alternatively, cisplatin may elicit a change in expression in the CDH1-negative tumor cells (i.e., a reduction in replication rate that contributes to cisplatin resistance and may lead to concomitant re-expression of CDH1). Although we cannot formally exclude this, we consider this unlikely, as the CDH1-negative compartments observed in RPM mice, which look indistinguishable from the CDH1-negative intrapulmonary metastasis in RPF mice, are initially effectively depleted by cisplatin treatment. Furthermore, we would expect that upon treatment with cisplatin, tumors in RPF mice would only very transiently become CDH1-positive. That is not what we observe. Whereas CDH1 expression in the setting of our autochthonous models seems to serve as a marker for chemotherapy resistance, this is not observed in cell lines or in PDX models of SCLC, where even the opposite is described (Drapkin et al., 2018, Allison Stewart et al., 2017). We have no conclusive explanation for this. Possibly, *in vitro* propagated cells or PDX lines still do not reflect the primary tumor, or the association of CDH1 expression with chemotherapy exposure is accidental. Human biopsies of SCLC show great variability of CDH1 expression (Semenova et al., 2016), but to determine its association with chemotherapy resistance will require a careful comparison of human SCLC biopsies that were obtained before and after chemotherapy.

However, our transcriptome and proteome analyses do provide further insight into this acquired chemotherapy resistance, such as a marked reduction in proliferation, as well as a pronounced shift in the metabolic program. In addition, whereas cisplatin-sensitive tumors show a clear pro-neuronal expression signature, resistant tumor cells show reduced expression of migration and neuronal differentiation-associated genes and an increase in epithelial differentiation proteins.

Analysis of RPM mice highlighted a broad topological and histopathological diversity of NE tumors that can arise in mouse lung. Of particular interest are tumors located in the bronchiolar and alveolar region. They represent well-differentiated NE lesions distinct from classical SCLC. They are also different from the lesions recently described by Huang et al. (2018), indicating that SCLC might include even more distinct subtypes. More peripherally located lesions have recently also been reported by Yang et al. (2018) in a mouse model of SCLC based on the inactivation of *Rb1*, *Trp53*, and *p130*. Future work is needed to reveal whether the tumors they described share common denominators with tumors we observed here and to what extent the different driver lesions engineered into these models are responsible for the distinct tumor characteristics. Because the alveolar lesions explored in more detail here were rare in untreated RPF mice, we could not collect pre-treatment alveolar lesions and therefore were unable to perform a direct comparison between treated and untreated cases. However, on the basis of histopathological evaluation of alveolar lesions in both RPF and RPM cohorts, they appeared unaffected by treatment, suggesting that they represent an intrinsically cisplatin-resistant NE tumor subtype. Comparison between Cis-RPF-Rep and Cis-RPF-Al, which are both cisplatin resistant, revealed for each unique metabolic and structural profiles that might aid in a search for corresponding tumor subtypes in patients and may be valuable for the design of therapeutic strategies against specific sub-populations of SCLC. For example, the alveolar lesions of RPF mice displayed increased expression of genes involved in redox reactions and drug detoxification, opening up the intriguing possibility of targeting these intrinsically cisplatin-resistant populations with additional oxidative stress and reactive oxygen species (ROS)-inducing agents.

In conclusion, using mouse models of SCLC has permitted us to carefully dissect SCLC tumor initiation, tumor heterogeneity, and drug responses, leading to the characterization of additional SCLC subtypes that exhibit differential expression patterns and drug-resistant profiles. What we observe in the mouse with regard to drug resistance aligns well with what is observed in the vast majority of human SCLC, with its initially good chemotherapy response followed by massive resistance. Given this similar behavior these mouse models can help us better understand the complexity and plasticity of SCLC and develop more effective therapies for this aggressive cancer type.

## STAR★Methods

### Key Resources Table

**Table:** 

REAGENT or RESOURCE	SOURCE	IDENTIFIER
**Antibodies**		

Synaptophysin, clone YE269	Abcam	Cat# ab32127; RRID: AB_2286949
E-Cadherin, clone 24E10	Cell Signaling Technology	Cat# 3195; RRID: AB_2291471
NFIB	Thermo Fisher Scientific	Cat# PA5-28299; RRID: AB_2545775
Calcitonin Gene Related Peptide (CGRP)	Sigma-Aldrich	Cat# C8198; RRID: AB_259091
Ki67	Abcam	Cat# ab15580; RRID: AB_443209
Anti-Cytokeratin, wide-spectrum/KWS	Agilent	Cat# Z0622; RRID: AB_2650434
Anti-phospho-Histone H3 (Thr3), clone JY325	Merck Millipore	Cat# 04-746; RRID: AB_1163442
Podoplanin	Abcam	Cat# ab11936; RRID: AB_298718
MASH1/ASCL1	BD Biosciences	Cat# 556604; RRID: AB_396479
ALDH1A1	Abcam	Cat# ab23375; RRID: AB_2224009
NEUROD1	Proteintech	Cat# 12081-1-AP
Phospho-Akt (Ser473)	Cell Signaling Technology	Cat# 4060; RRID: AB_2315049
Anti-4E-BP1, phospho (Thr37 / Thr46)	Cell Signaling Technology	Cat# 2855; RRID: AB_560835

**Bacterial and Virus Strains**		

Ad5-CMV-Cre	Viral Vector Core Facility, University of IOWA Health Care	N/A

**Chemicals, Peptides, and Recombinant Proteins**		

Cyclosporin A	Sigma-Aldrich	Cat# 30024
Cisplatin	Accord Healthcare Ltd	Dutch drug database ZI# 15683354
Neo-clear®	Merck Millipore	Cat# 109843
Sequencing Grade Modified Trypsin	Promega	Cat# V5111

**Deposited Data**		

Mass spectrometry proteomics data	This study	ProteomeXchange: PXD010680
RNA-seq data	This study	European Nucleotide Archive: PRJEB28270

**Experimental Models: Organisms/Strains**		

Mouse: Rb1^flox/flox^;Trp53^flox/flox^	Meuwissen et al., 2003	N/A
Mouse: Rb1^flox/flox^;Trp53^flox/flox^;CAG < Lox66Mycl-LucLox71 >	Huijbers et al., 2014	N/A
Mouse: Rb1^flox/flox^;Trp53^flox/flox^;CAG < Lox66Nfib-LucLox71 >	Semenova et al., 2016	N/A

**Software and Algorithms**		

Living Image acquisition and analysis software	PerkinElmer	N/A
AxioVision 4 software	Carl Zeiss Vision	N/A
MaxQuant version 1.5.4.1	Cox and Mann, 2008	http://www.coxdocs.org
TopHat, version 2.1.0	Kim et al., 2013	https://ccb.jhu.edu/software/tophat/index.shtml
GraphPad Prism, versions 6 and 7.04	GraphPad Software	www.graphpad.com
Perseus software (version 1.6.1.3)	Tyanova et al., 2016	http://www.coxdocs.org
GSEA 3.0	Mootha et al., 2003, Subramanian et al., 2005	www.broadinstitute.org/gsea
STRING tool (version 10.5)	Szklarczyk et al., 2017	www.string-db.org
Cytoscape (version 3.4.0)	Shannon et al., 2003	www.cytoscape.org
DAVID tool (version 6.8)	Huang et al., 2009	david.ncifcrf.gov

**Other**		

IVIS Spectrum *In Vivo* Imaging System	PerkinElmer	N/A
Zeiss Axioskop2 Plus microscope	Carl Zeiss Microscopy	N/A
Zeiss AxioCam HRc digital camera	Carl Zeiss Vision	N/A
Q Exactive Hybrid Quadrupole-Orbitrap Mass Spectrometer	Thermo Fisher Scientific	N/A
Ultimate 3000 nanoLC	Thermo Fisher Scientific	N/A
HiSeq 2000 Sequencing System	Illumina	N/A

### Contact for Reagent and Resource Sharing

Further information and requests for resources and reagents should be directed to and will be fulfilled by the Lead Contact, Anton Berns (a.berns@nki.nl).

### Experimental Model and Subject Details

#### *In Vivo* Mouse Studies

All animals were maintained on an FVB background (backcrossed from strains generated from 129 Ola ESCs). Either RPM or RPF mice were randomly assigned to the experimental cisplatin or vehicle group, 5 weeks upon receiving the adenoviral injection. On average, mice were aged 11.3 weeks (SD ± 4.5) at virus injection. Male and female mice were represented equally in the experimental cohorts and were group-housed (maximum 4 mice per cage), in individually ventilated cages (IVC) with standard enrichment. The study was performed in accordance with the Dutch and European regulations on care and protection of laboratory animals. All animal experiments were approved by the institute’s Animal Ethical Committee.

### Method Details

#### Lung tumor induction

Mice from the different cohorts were treated with Cyclosporin A (Sigma-Aldrich) orally in the drinking water 1 week prior to adenovirus administration and 2-3 weeks following infection. Viral Ad5-CMV-Cre particles (20 μl, 1 × 10^9^; Viral Vector Core Facility, University of IOWA Health Care) were injected intratracheally. Mice were monitored daily for signs of illness and culled upon respiratory distress or excessive weight loss (> 20% of initial weight).

#### Imaging of tumors

*In vivo* bioluminescence imaging was performed and quantified as described by Hsieh et al. (2005) on a cryogenically cooled IVIS Spectrum system using Living Image acquisition and analysis software (both PerkinElmer). Luciferase units are photons/second x cm^2^ x sr.

#### Cisplatin treatment

Tumor growth was monitored via weekly bioluminescence imaging. Treatment was initiated when signal within the thorax area reached 1 × 10^5^ relative luciferase units. Animals were injected with cisplatin (Accord Healthcare Ltd) at 6 mg/kg i.v. once every two weeks.

#### Histology and immunohistochemistry

Animals were sacrificed when they acquired respiratory distress. Tissues and organs were collected and fixed in EAF fixative (ethanol/acetic acid/formaldehyde/saline at 40:5:10:45 v/v) and embedded in paraffin. Sections were prepared at 2 μm thickness from the paraffin blocks and stained with hematoxylin and eosin (HE) according to standard procedures. For immunohistochemistry (IHC), 4 μm-thick sections were made on which the following antibodies were applied: Synaptophysin/SYP (Abcam, ab32127), E-cadherin/CDH1 (Cell Signaling Technology, 3195), NFIB (Thermo Fisher Scientific, PA5-28299), CGRP (Sigma-Aldrich, C8198), Ki67 (Abcam, ab15580), keratin wide-spectrum/KWS (Agilent, Z0622), phospho-histone H3/p-HH3 (Millipore, 04-746), Podoplanin/PDPN (Abcam, ab11936), ASCL1 (BD Biosciences, 556604), ALDH1A1 (Abcam, ab23375), NEUROD1 (Proteintech, 12081-1-ap), phospho-AKT (Cell Signaling Technology, 4060) and phospho-4EBP1 (Cell Signaling Technology, 2855). The sections were reviewed with a Zeiss Axioskop2 Plus microscope (Carl Zeiss Microscopy) and images were captured with a Zeiss AxioCam HRc digital camera and processed with AxioVision 4 software (both from Carl Zeiss Vision).

#### Tandem mass-spectrometry

EAF sections of 10 μm thickness were cut from the paraffin blocks, with reference slides for HE- and CDH1-staining being taken at regular intervals during cutting. Blank EAF-fixed sections (10 × 10 μm) of mouse lung were deparaffinized using Neo-clear® (3 times 5 minutes) and rehydrated by subsequent 3-minute incubations in 100%, 80% (v/v) and 70% (v/v) ethanol, and finally water. Subsequently, tumor areas were scraped from the slide using a needle, according to the demarcated HE- and CDH1-stained reference slides. Scraped tumor tissues were lysed in Tris-HCl buffer (pH 8.0), vortexed, sonicated and, after the addition of 2% (w/v) SDS, heat-incubated for 90 min while shaking (1400 rpm). Cell debris was removed by centrifugation for 20 minutes at 16.000 x g, and resulting clear lysate was supplemented with 4X LDS sample buffer and DTT for subsequent SDS-polyacrylamide gel electrophoresis.

Protein lysates were separated on pre-cast 4%–12% gradient gels using the NuPAGE SDS-PAGE system (Invitrogen, Carlsbad, CA). Gels were fixed in 50% ethanol/3% phosphoric acid solution, stained with Coomassie brilliant blue G-250 and then washed and dehydrated in 50 mM ammonium bicarbonate (ABC) (once) and 50 mM ABC/50% acetonitrile (ACN) twice. Gel lanes were cut into five bands, with each band sliced further into approximately 1 mm^3^ cubes. Gel cubes were washed and dehydrated once in 50 mM ABC and twice in 50 mM ABC/50% ACN. Subsequently, gel cubes were reduced in 10 mM DTT/50 mM ABC at 56°C for 1 h, the supernatant was removed, and gel cubes were alkylated in 50 mM iodoacetamide/50 mM ABC for 45 min at room temperature in the dark. Next, gel cubes were washed with 50 mM ABC/50% ACN, dried in a vacuum centrifuge at 50°C for 10 min and covered with trypsin solution (Promega, 6.25 ng/ml in 50 mM ABC). Following rehydration with trypsin solution and removal of excess trypsin, gel cubes were covered with 50 mM ABC and incubated overnight at 25°C. Peptides were extracted from the gel cubes with 1% formic acid (FA) (once) and 5% FA/50% ACN (twice). All extracts were pooled and stored at −20°C until use. Prior to LC-MS, the extracts were concentrated in a vacuum centrifuge at 50°C, and volumes were adjusted to 50 μL with 0.05% FA, filtered through a 0.45 μm spin filter, and transferred to LC autosampler vials.

Peptides (5 μl) were separated by an Ultimate 3000 nanoLC-MS/MS system (Thermo Fisher, Bremen, Germany), equipped with a 20 cm × 75 μm ID fused silica column custom packed with 1.9 μm 120 A° ReproSil Pur C18 aqua (Dr Maisch GMBH, Ammerbuch-Entringen, Germany). After injection, peptides were trapped at 6 μl/min on a 10 mm × 100 μm ID trap column packed with 5 μm 120 A° ReproSil Pur C18 aqua in 0.05% FA. Peptides were separated at 300 nl/min in a 10%–40% gradient (buffer A: 0.5% acetic acid, buffer B: 80% ACN, 0.5% acetic acid) in 60 min (90-min inject-to-inject). Eluting peptides were ionized at a potential of +2 kVa into a Q Exactive mass spectrometer (Thermo Fisher, Bremen, Germany). Intact masses were measured at resolution 70,000 (at m/z 200) in the orbitrap using an AGC target value of 3E6 charges. The top 10 peptide signals (charge-states 2+ and higher) were submitted to MS/MS in the HCD (higher-energy collision) cell (1.6 amu isolation width, 25% normalized collision energy). MS/MS spectra were acquired at resolution 17,500 (at m/z 200) in the orbitrap using an AGC target value of 1E6 charges, a maxIT of 60 ms, and an underfill ratio of 0.1%. Dynamic exclusion was applied with a repeat count of 1 and an exclusion time of 30 s.

MS/MS spectra were searched against the Swissprot *Mus musculus* reference proteome FASTA file (release August 2017, 25052 entries, canonical and isoforms) using MaxQuant version 1.5.4.1 (Cox and Mann, 2008). Enzyme specificity was set to trypsin, and up to two missed cleavages were allowed. Cysteine carbamidomethylation (Cys, +57.021464 Da) was treated as fixed modification and methionine oxidation (Met, +15.994915 Da) and N-terminal acetylation (N-terminal, +42.010565 Da) as variable modifications. Peptide precursor ions were searched with a maximum mass deviation of 4.5 ppm and fragment ions with a maximum mass deviation of 20 ppm. Peptide and protein identifications were filtered at an FDR of 1% using the decoy database strategy. The minimal peptide length was 7 amino acids. Proteins that could not be differentiated based on MS/MS spectra alone were grouped into protein groups (default MaxQuant settings). Searches were performed with the label-free quantification option selected. Proteins were quantified by spectral counting (Liu et al., 2004).

#### RNA sequencing

RNA was isolated using Trizol, and cDNA libraries were sequenced on an Illumina HiSeq2000 to obtain 65-bp single-end sequence reads. Reads were aligned to the mm10 mouse reference genome using TopHat (Kim et al., 2013), and gene counts were obtained using HTSeq (Anders et al., 2015).

### Quantification and Statistical Analysis

#### Normalization and statistical analyses

For proteomics data, raw spectral counts were normalized on the sum of spectral counts for all identified proteins in a particular sample, relative to the average sample sum determined with all samples. To find statistically significant differences in normalized counts between sample groups, we applied the beta-binomial test (Pham et al., 2010), which takes into account within-sample and between-sample variation. For both normalization and differential analysis of RNA-seq data, DESeq2 (Love et al., 2014) was used. All downstream analyses were performed in R using the Bioconductor framework. The statistical methods used as well as the p values defining significance are stated in all legends of figures referencing this data.

#### Survival analysis

Kaplan-Meier survival curves were analyzed using the log-rank test. All p values were calculated using a nonparametric Mann-Whitney test (statistical analyses were performed by GraphPad Prism, version 6).

#### Data visualization

Heatmaps were generated using Perseus software, version 1.6.1.3 (Tyanova et al., 2016), using z-score by column of normalized spectral or read counts for protein and RNA data, respectively (euclidean distance, complete linkage). Expression plots were made using GraphPad Prism (version 7.04), volcano plots using R (version 3.4.1). Overlapping genes were identified using the Venny tool (version 2.1). For gene set enrichment analysis using GSEA 3.0 (Mootha et al., 2003, Subramanian et al., 2005), a rank metric score based on combined fold-change and p values was used as input. Normalized enrichment score (NES) was used as the magnitude of enrichment, FDR q-value as a weighted measure of statistical significance. Network analysis was performed using STRING tool, version 10.5 (Szklarczyk et al., 2015) and visualized using Cytoscape, version 3.4.0 (Shannon et al., 2003), employing Cytoscape MCL cluster plugin. Gene ontology was analyzed using DAVID tool, version 6.8 (Huang et al., 2009).

### Data and Software Availability

The accession number for the mass spectrometry proteomics data reported in this paper is ProteomeXchange: PXD010680. The accession number for the RNA-seq data reported in this paper is European Nucleotide Archive (ENA): PRJEB28270.

## Supplemental Information

Document S1. Figures S1–S6

Table S1, Details of Cisplatin Treatment and Survival, Related to Figure 3

Table S2, RNA Expression Data, Related to Figures 4 and 5

Table S3, Protein Expression Data, Related to Figures 4 and 5

Table S4, GSEA Core Enrichment Genes, Related to Figure 4

Document S6. Article plus Supplemental Information

## References

[bib1] Allison Stewart, C., Tong, P., Cardnell, R.J., Sen, T., Li, L., Gay, C.M., Masrorpour, F., Fan, Y., Bara, R.O., Feng, Y., et al. (2017). Dynamic variations in epithelial-to-mesenchymal transition (EMT), ATM, and SLFN11 govern response to PARP inhibitors and cisplatin in small cell lung cancer. Oncotarget 8, 28575-28587. 10.18632/oncotarget.15338 PMC5438673 28212573

[bib2] Anders, S., Pyl, P.T., and Huber, W. (2015). HTSeq-a Python framework to work with high-throughput sequencing data. Bioinformatics 31, 166-169. 10.1093/bioinformatics/btu638 PMC4287950 25260700

[bib3] Borromeo, M.D., Savage, T.K., Kollipara, R.K., He, M., Augustyn, A., Osborne, J.K., Girard, L., Minna, J.D., Gazdar, A.F., Cobb, M.H., and Johnson, J.E. (2016). ASCL1 and NEUROD1 reveal heterogeneity in pulmonary neuroendocrine tumors and regulate distinct genetic programs. Cell Rep. 16, 1259-1272. 10.1016/j.celrep.2016.06.081 PMC4972690 27452466

[bib4] Calbo, J., van Montfort, E., Proost, N., van Drunen, E., Beverloo, H.B., Meuwissen, R., and Berns, A. (2011). A functional role for tumor cell heterogeneity in a mouse model of small cell lung cancer. Cancer Cell 19, 244-256. 10.1016/j.ccr.2010.12.021 21316603

[bib5] Cox, J., and Mann, M. (2008). MaxQuant enables high peptide identification rates, individualized p.p.b.-range mass accuracies and proteome-wide protein quantification. Nat. Biotechnol. 26, 1367-1372. 10.1038/nbt.1511 19029910

[bib6] De Santis, G., Miotti, S., Mazzi, M., Canevari, S., and Tomassetti, A. (2009). E-cadherin directly contributes to PI3K/AKT activation by engaging the PI3K-p85 regulatory subunit to adherens junctions of ovarian carcinoma cells. Oncogene 28, 1206-1217. 10.1038/onc.2008.470 19151754

[bib7] Denny, S.K., Yang, D., Chuang, C.-H.H., Brady, J.J., Lim, J.S.S., Gruner, B.M., Chiou, S.-H.H., Schep, A.N., Baral, J., Hamard, C., et al. (2016). Nfib promotes metastasis through a widespread increase in chromatin accessibility. Cell 166, 328-342. 10.1016/j.cell.2016.05.052 PMC5004630 27374332

[bib8] Doerr, F., George, J., Schmitt, A., Beleggia, F., Rehkamper, T., Hermann, S., Walter, V., Weber, J.-P., Thomas, R.K., Wittersheim, M., et al. (2017). Targeting a non-oncogene addiction to the ATR/CHK1 axis for the treatment of small cell lung cancer. Sci. Rep. 7, 15511. 10.1038/s41598-017-15840-5 PMC5686113 29138515

[bib9] Drapkin, B.J., George, J., Christensen, C.L., Mino-Kenudson, M., Dries, R., Sundaresan, T., Phat, S., Myers, D.T., Zhong, J., Igo, P., et al. (2018). Genomic and functional fidelity of small cell lung cancer patient-derived xenografts. Cancer Discov. 8, 600-615. 10.1158/2159-8290.CD-17-0935 PMC6369413 29483136

[bib10] Farago, A.F., and Keane, F.K. (2018). Current standards for clinical management of small cell lung cancer. Transl. Lung Cancer Res. 7, 69-79. 10.21037/tlcr.2018.01.16 PMC5835595 29535913

[bib11] Fernandez, H.R., Gadre, S.M., Tan, M., Graham, G.T., Mosaoa, R., Ongkeko, M.S., Kim, K.A., Riggins, R.B., Parasido, E., Petrini, I., et al. (2018). The mitochondrial citrate carrier, SLC25A1, drives stemness and therapy resistance in non-small cell lung cancer. Cell Death Differ. 25, 1239-1258. 10.1038/s41418-018-0101-z PMC6030199 29651165

[bib12] Gardner, E.E., Lok, B.H., Schneeberger, V.E., Desmeules, P., Miles, L.A., Arnold, P.K., Ni, A., Khodos, I., de Stanchina, E., Nguyen, T., et al. (2017). Chemosensitive relapse in small cell lung cancer proceeds through an EZH2-SLFN11 axis. Cancer Cell 31, 286-299. 10.1016/j.ccell.2017.01.006 PMC5313262 28196596

[bib13] Gazdar, A.F., Carney, D.N., Russell, E.K., Sims, H.L., Baylin, S.B., Bunn, P.A., Jr., Guccion, J.G., and Minna, J.D. (1980). Establishment of continuous, clonable cultures of small-cell carcinoma of lung which have amine precursor uptake and decarboxylation cell properties. Cancer Res. 40, 3502-3507. 6108156

[bib14] Gazdar, A.F., Savage, T.K., Johnson, J.E., Berns, A., Sage, J., Linnoila, R.I., MacPherson, D., McFadden, D.G., Farago, A., Jacks, T., et al. (2015). The comparative pathology of genetically engineered mouse models for neuroendocrine carcinomas of the lung. J. Thorac. Oncol. 10, 553-564. 10.1097/JTO.0000000000000459 PMC4523224 25675280

[bib15] Gazdar, A.F., Bunn, P.A., and Minna, J.D. (2017). Small-cell lung cancer: what we know, what we need to know and the path forward. Nat. Rev. Cancer 17, 725-737. 10.1038/nrc.2017.87 29077690

[bib16] George, J., Lim, J.S., Jang, S.J., Cun, Y., Ozretić, L., Kong, G., Leenders, F., Lu, X., Fernandez-Cuesta, L., Bosco, G., et al. (2015). Comprehensive genomic profiles of small cell lung cancer. Nature 524, 47-53. 10.1038/nature14664 PMC4861069 26168399

[bib17] George, J., Walter, V., Peifer, M., Alexandrov, L.B., Seidel, D., Leenders, F., Maas, L., Muller, C., Dahmen, I., Delhomme, T.M., et al. (2018). Integrative genomic profiling of large-cell neuroendocrine carcinomas reveals distinct subtypes of high-grade neuroendocrine lung tumors. Nat. Commun. 9, 1048. 10.1038/s41467-018-03099-x PMC5849599 29535388

[bib18] Hellmann, M.D., Callahan, M.K., Awad, M.M., Calvo, E., Ascierto, P.A., Atmaca, A., Rizvi, N.A., Hirsch, F.R., Selvaggi, G., Szustakowski, J.D., et al. (2018). Tumor mutational burden and efficacy of nivolumab monotherapy and in combination with ipilimumab in small-cell lung cancer. Cancer Cell 33, 853-861.e4. 10.1016/j.ccell.2018.04.001 PMC6750707 29731394

[bib19] Hsieh, C.-L., Xie, Z., Liu, Z.-Y., Green, J.E., Martin, W.D., Datta, M.W., Yeung, F., Pan, D., and Chung, L.W.K. (2005). A luciferase transgenic mouse model: visualization of prostate development and its androgen responsiveness in live animals. J. Mol. Endocrinol. 35, 293-304. 10.1677/jme.1.01722 16216910

[bib20] Huang, W., Sherman, B.T., and Lempicki, R.A. (2009). Systematic and integrative analysis of large gene lists using DAVID bioinformatics resources. Nat. Protoc. 4, 44-57. 10.1038/nprot.2008.211 19131956

[bib21] Huang, Y.-H., Klingbeil, O., He, X.-Y., Wu, X.S., Arun, G., Lu, B., Somerville, T.D.D., Milazzo, J.P., Wilkinson, J.E., Demerdash, O.E., et al. (2018). POU2F3 is a master regulator of a tuft cell-like variant of small cell lung cancer. Genes Dev. 32, 915-928. 10.1101/gad.314815.118 PMC6075037 29945888

[bib22] Huijbers, I.J., Bin Ali, R., Pritchard, C., Cozijnsen, M., Kwon, M.C., Proost, N., Song, J.Y., de Vries, H., Badhai, J., Sutherland, K., et al. (2014). Rapid target gene validation in complex cancer mouse models using re-derived embryonic stem cells. EMBO Mol. Med. 6, 212-225. 10.1002/emmm.201303297 PMC3927956 24401838

[bib23] Kim, D., Pertea, G., Trapnell, C., Pimentel, H., Kelley, R., and Salzberg, S.L. (2013). TopHat2: accurate alignment of transcriptomes in the presence of insertions, deletions and gene fusions. Genome Biol. 14, R36. 10.1186/gb-2013-14-4-r36 PMC4053844 23618408

[bib24] Koinis, F., Kotsakis, A., and Georgoulias, V. (2016). Small cell lung cancer (SCLC): no treatment advances in recent years. Transl. Lung Cancer Res. 5, 39-50. 10.3978/j.issn.2218-6751.2016.01.03 PMC4758968 26958492

[bib25] Kumar, V.H.S., Chaker El Khoury, J., Gronostajski, R., Wang, H., Nielsen, L., and Ryan, R.M. (2017). Nfib hemizygous mice are protected from hyperoxic lung injury and death. Physiol. Rep. 5, 1-11. 10.14814/phy2.13398 PMC5582271 28830981

[bib26] Kuo, C.S., and Krasnow, M.A. (2015). Formation of a neurosensory organ by epithelial cell slithering. Cell 163, 394-405. 10.1016/j.cell.2015.09.021 PMC4597318 26435104

[bib27] Lim, J.S., Ibaseta, A., Fischer, M.M., Cancilla, B., O’Young, G., Cristea, S., Luca, V.C., Yang, D., Jahchan, N.S., Hamard, C., et al. (2017). Intratumoural heterogeneity generated by Notch signalling promotes small-cell lung cancer. Nature 545, 360-364. 10.1038/nature22323 PMC5776014 28489825

[bib28] Liu, H., Sadygov, R.G., and Yates, J.R., 3rd (2004). A model for random sampling and estimation of relative protein abundance in shotgun proteomics. Anal. Chem. 76, 4193-4201. 10.1021/ac0498563 15253663

[bib29] Love, M.I., Huber, W., and Anders, S. (2014). Moderated estimation of fold change and dispersion for RNA-seq data with DESeq2. Genome Biol. 15, 550. 10.1186/s13059-014-0550-8 PMC4302049 25516281

[bib30] MacDonagh, L., Gallagher, M.F., Ffrench, B., Gasch, C., Breen, E., Gray, S.G., Nicholson, S., Leonard, N., Ryan, R., Young, V., et al. (2017). Targeting the cancer stem cell marker, aldehyde dehydrogenase 1, to circumvent cisplatin resistance in NSCLC. Oncotarget 8, 72544-72563. 10.18632/oncotarget.19881 PMC5641151 29069808

[bib31] Meuwissen, R., Linn, S.C., Linnoila, R.I., Zevenhoven, J., Mooi, W.J., and Berns, A. (2003). Induction of small cell lung cancer by somatic inactivation of both Trp53 and Rb1 in a conditional mouse model. Cancer Cell 4, 181-189. 10.1016/s1535-6108(03)00220-4 14522252

[bib32] Mezzadra, R., de Bruijn, M., Jae, L.T., Gomez-Eerland, R., Duursma, A., Scheeren, F.A., Brummelkamp, T.R., and Schumacher, T.N. (2019). SLFN11 can sensitize tumor cells towards IFN-γ-mediated T cell killing. PLoS ONE 14, e0212053. 10.1371/journal.pone.0212053 PMC6372190 30753225

[bib33] Mollaoglu, G., Guthrie, M.R., Bohm, S., Bragelmann, J., Can, I., Ballieu, P.M., Marx, A., George, J., Heinen, C., Chalishazar, M.D., et al. (2017). MYC drives progression of small cell lung cancer to a variant neuroendocrine subtype with vulnerability to aurora kinase inhibition. Cancer Cell 31, 270-285. 10.1016/j.ccell.2016.12.005 PMC5310991 28089889

[bib34] Mootha, V.K., Lindgren, C.M., Eriksson, K.-F., Subramanian, A., Sihag, S., Lehar, J., Puigserver, P., Carlsson, E., Ridderstrale, M., Laurila, E., et al. (2003). PGC-1α-responsive genes involved in oxidative phosphorylation are coordinately downregulated in human diabetes. Nat. Genet. 34, 267-273. 10.1038/ng1180 12808457

[bib35] Morandi, A., and Indraccolo, S. (2017). Linking metabolic reprogramming to therapy resistance in cancer. Biochim Biophys Acta Rev Cancer 1868, 1-6. 10.1016/j.bbcan.2016.12.004 28065746

[bib36] Nagel, R., Avelar, A.T., Aben, N., Proost, N., van de Ven, M., van der Vliet, J., Cozijnsen, M., de Vries, H., Wessels, L.F.A., and Berns, A. (2019). Inhibition of the replication stress response is a synthetic vulnerability in SCLC that acts synergistically in combination with cisplatin. Mol. Cancer Ther. 18, 762-770. 10.1158/1535-7163.MCT-18-0972 PMC6451635 30872379

[bib37] Niederst, M.J., Sequist, L.V., Poirier, J.T., Mermel, C.H., Lockerman, E.L., Garcia, A.R., Katayama, R., Costa, C., Ross, K.N., Moran, T., et al. (2015). RB loss in resistant EGFR mutant lung adenocarcinomas that transform to small-cell lung cancer. Nat. Commun. 6, 6377. 10.1038/ncomms7377 PMC4357281 25758528

[bib38] Park, K.-S., Liang, M.-C., Raiser, D.M., Zamponi, R., Roach, R.R., Curtis, S.J., Walton, Z., Schaffer, B.E., Roake, C.M., Zmoos, A.-F., et al. (2011). Characterization of the cell of origin for small cell lung cancer. Cell Cycle 10, 2806-2815. 10.4161/cc.10.16.17012 PMC3219544 21822053

[bib39] Pham, T.V., Piersma, S.R., Warmoes, M., and Jimenez, C.R. (2010). On the beta-binomial model for analysis of spectral count data in label-free tandem mass spectrometry-based proteomics. Bioinformatics 26, 363-369. 10.1093/bioinformatics/btp677 20007255

[bib40] Pozo, K., Minna, J.D., and Johnson, J.E. (2018). Identifying a missing lineage driver in a subset of lung neuroendocrine tumors. Genes Dev. 32, 865-867. 10.1101/gad.316943.118 PMC6075039 29967289

[bib41] Rossi, A., Tay, R., Chiramel, J., Prelaj, A., and Califano, R. (2018). Current and future therapeutic approaches for the treatment of small cell lung cancer. Expert Rev. Anticancer Ther. 18, 473-486. 10.1080/14737140.2018.1453361 29544351

[bib42] Semenova, E.A., Nagel, R., and Berns, A. (2015). Origins, genetic landscape, and emerging therapies of small cell lung cancer. Genes Dev. 29, 1447-1462. 10.1101/gad.263145.115 PMC4526731 26220992

[bib43] Semenova, E.A., Kwon, M.C., Monkhorst, K., Song, J.Y., Bhaskaran, R., Krijgsman, O., Kuilman, T., Peters, D., Buikhuisen, W.A., Smit, E.F., et al. (2016). Transcription factor NFIB is a driver of small cell lung cancer progression in mice and marks metastatic disease in patients. Cell Rep. 16, 631-643. 10.1016/j.celrep.2016.06.020 PMC4956617 27373156

[bib44] Sen, T., Tong, P., Stewart, C.A., Cristea, S., Valliani, A., Shames, D.S., Redwood, A.B., Fan, Y.H., Li, L., Glisson, B.S., et al. (2017). CHK1 inhibition in small-cell lung cancer produces single-agent activity in biomarker-defined disease subsets and combination activity with cisplatin or olaparib. Cancer Res. 77, 3870-3884. 10.1158/0008-5472.CAN-16-3409 PMC5563854 28490518

[bib45] Sequist, L.V., Waltman, B.A., Dias-Santagata, D., Digumarthy, S., Turke, A.B., Fidias, P., Bergethon, K., Shaw, A.T., Gettinger, S., Cosper, A.K., et al. (2011). Genotypic and histological evolution of lung cancers acquiring resistance to EGFR inhibitors. Sci. Transl. Med. 3, 75ra26. 10.1126/scitranslmed.3002003 PMC3132801 21430269

[bib46] Shannon, P., Markiel, A., Ozier, O., Baliga, N.S., Wang, J.T., Ramage, D., Amin, N., Schwikowski, B., and Ideker, T. (2003). Cytoscape: a software environment for integrated models of biomolecular interaction networks. Genome Res. 13, 2498-2504. 10.1101/gr.1239303 PMC403769 14597658

[bib47] Shue, Y.T., Lim, J.S., and Sage, J. (2018). Tumor heterogeneity in small cell lung cancer defined and investigated in pre-clinical mouse models. Transl. Lung Cancer Res. 7, 21-31. 10.21037/tlcr.2018.01.15 PMC5835592 29535910

[bib48] Subramanian, A., Tamayo, P., Mootha, V.K., Mukherjee, S., Ebert, B.L., Gillette, M.A., Paulovich, A., Pomeroy, S.L., Golub, T.R., Lander, E.S., and Mesirov, J.P. (2005). Gene set enrichment analysis: a knowledge-based approach for interpreting genome-wide expression profiles. Proc. Natl. Acad. Sci. U S A 102, 15545-15550. 10.1073/pnas.0506580102 PMC1239896 16199517

[bib49] Sutherland, K.D., Proost, N., Brouns, I., Adriaensen, D., Song, J.Y., and Berns, A. (2011). Cell of origin of small cell lung cancer: inactivation of Trp53 and Rb1 in distinct cell types of adult mouse lung. Cancer Cell 19, 754-764. 10.1016/j.ccr.2011.04.019 21665149

[bib50] Szklarczyk, D., Franceschini, A., Wyder, S., Forslund, K., Heller, D., Huerta-Cepas, J., Simonovic, M., Roth, A., Santos, A., Tsafou, K.P., et al. (2015). STRING v10: protein-protein interaction networks, integrated over the tree of life. Nucleic Acids Res. 43, D447-D452. 10.1093/nar/gku1003 PMC4383874 25352553

[bib51] Szklarczyk, D., Morris, J.H., Cook, H., Kuhn, M., Wyder, S., Simonovic, M., Santos, A., Doncheva, N.T., Roth, A., Bork, P., et al. (2017). The STRING database in 2017: quality-controlled protein-protein association networks, made broadly accessible. Nucleic Acids Res. 45, D362-D368. 10.1093/nar/gkw937 PMC5210637 27924014

[bib52] Tyanova, S., Temu, T., Sinitcyn, P., Carlson, A., Hein, M.Y., Geiger, T., Mann, M., and Cox, J. (2016). The Perseus computational platform for comprehensive analysis of (prote)omics data. Nat. Methods 13, 731-740. 10.1038/nmeth.3901 27348712

[bib53] Ujhazy, P., and Lindwasser, O.W. (2018). Small cell lung cancer: updates and new concepts. Transl. Lung Cancer Res. 7, 1-3. 10.21037/tlcr.2018.02.01 PMC5835593 29535908

[bib55] Wu, N., Jia, D., Ibrahim, A.H., Bachurski, C.J., Gronostajski, R.M., and MacPherson, D. (2016). NFIB overexpression cooperates with Rb/p53 deletion to promote small cell lung cancer. Oncotarget 7, 57514-57524. 10.18632/oncotarget.11583 PMC5295369 27613844

[bib56] Yang, G., Sau, C., Lai, W., Cichon, J., and Li, W. (2015). Small cell lung cancer: can recent advances in biology and molecular biology be translated into improved outcomes? J. Thorac. Oncol. 344, 1173-1178. 10.1016/j.jtho.2016.01.012 PMC4836290 26829312

[bib57] Yang, D., Denny, S.K., Greenside, P.G., Chaikovsky, A.C., Brady, J.J., Ouadah, Y., Granja, J.M., Jahchan, N.S., Lim, J.S., Kwok, S., et al. (2018). Intertumoral heterogeneity in SCLC is influenced by the cell type of origin. Cancer Discov. 8, 1316-1331. 10.1158/2159-8290.CD-17-0987 PMC6195211 30228179

[bib58] Zhang, W., Girard, L., Zhang, Y.-A., Haruki, T., Papari-Zareei, M., Stastny, V., Ghayee, H.K., Pacak, K., Oliver, T.G., Minna, J.D., and Gazdar, A.F. (2018). Small cell lung cancer tumors and preclinical models display heterogeneity of neuroendocrine phenotypes. Transl. Lung Cancer Res. 7, 32-49. 10.21037/tlcr.2018.02.02 PMC5835590 29535911

[bib59] Zhao, X., McCutcheon, J.N., Kallakury, B., Chahine, J.J., Pratt, D., Raffeld, M., Chen, Y., Wang, C., and Giaccone, G. (2018). Combined small cell carcinoma of the lung: is it a single entity? J. Thorac. Oncol. 13, 237-245. 10.1016/j.jtho.2017.10.010 PMC8057113 29101056

