# Supplementary material for: Tumor Heterogeneity Underlies Differential Cisplatin Sensitivity in Mouse Models of Small-Cell Lung Cancer
Source: Cell Rep. 2019 Jun 11;27(11):3345–3358.e4. doi: 10.1016/j.celrep.2019.05.057 (PMC6581744; doi:10.1016/j.celrep.2019.05.057)
Supplement: Document S1. Figures S1–S6 [file mmc1.pdf]

Cell Reports, Volume *27*

Supplemental Information

Tumor Heterogeneity Underlies Differential Cisplatin Sensitivity in Mouse Models of Small-Cell Lung Cancer

Franziska Böttger, Ekaterina A. Semenova, Ji-Ying Song, Giustina Ferone, Jan van der Vliet, Miranda Cozijnsen, Rajith Bhaskaran, Lorenzo Bombardelli, Sander R. Piersma, Thang V. Pham, Connie R. Jimenez, and Anton Berns

Figure S1. Related to Figures 1 and 2

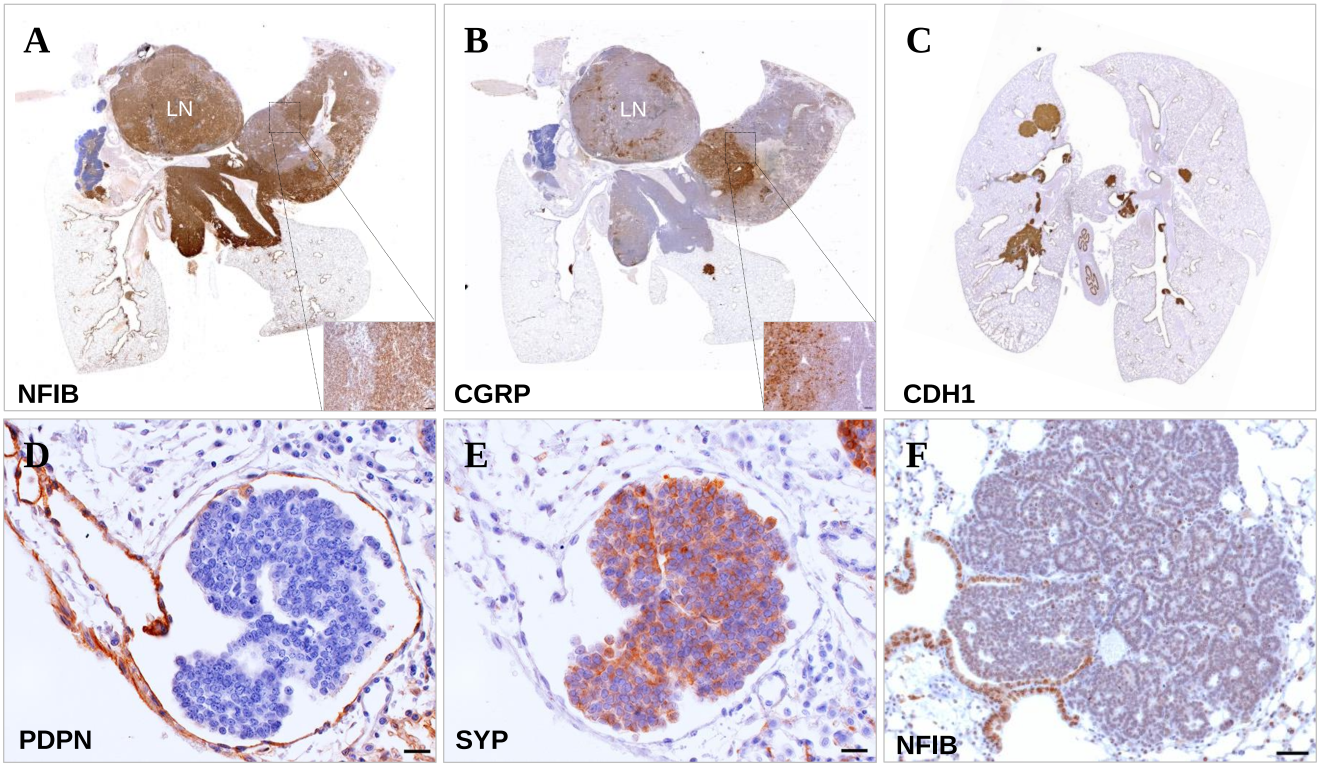

Figure S1. Related to Figures 1 and 2.
(A), (B) NFIB and CGRP staining of the lung of RP mouse, respectively. Inset shows an area of the central tumor with two tumor populations in close proximity, scale bar 50 µm. **(C)** CDH1 staining of a representative lung of a tumor bearing mouse taken at an early time point (14 weeks) after tumor induction showing positive signal for all primary lesions. **(D), (E)** Podoplanin and synaptophysin staining respectively, showing NE cells within a lymph vessel. **(F)** NFIB stainig of an alveolar lesion. Scale bar for (D), (E), 20 µm. Scale bar for (F), 50 µm.

Figure S2. Related to Figure 3

A

	ASCL-1	NEUROD-1	NFIB	p-AKT	p-4EBP1	CGRP	CDH1
**V-RPM mice**							
16ESE043	65%	20%	65%	15%	70%	50%	35%
16ESE047	90%	15%	5%	25%	40%	70%	70%
16ESE057	90%	20% (very weak)	15%	5%	50%	50%	90%
16IUB909	70%	10%	50%	15%	50%	50%	90%
17IUA054	85%	10% (weak)	60%	10%	85%	30%	30%
**Cis-RPM mice**							
16ESE040	90%	5%	5%	50%	60%	85%	95%
16ESE048	85%	20% (weak)	30%	20%	40%	80%	95%
16ESE052	70%	5%	10%	10%	35%	20%	95%
16ESE053	70%	20% (weak)	65%	15%	40%	45%	50%
16ESE054	75%	20%	15%	35%	70%	70%	90%
**V-RPF mice**							
17ESE001	90%	5% (weak)	35%	20%	70%	25%	50%
17ESE005	85%	5% (weak)	20%	10%	70%	25%	30%
17ESE006	90%	10%	20% (weak)	10%	85%	30%	15%
17ESE008	95%	10%	30%	15%	65%	60%	30%
17ESE011	80%	30%	50%	5%	70%	35%	40%
**Cis-RPF mice**							
17ESE002	85%	30% (weak)	60%	10%	70%	45%	99%
17ESE003	80%	20%	50%	25%	50%	60%	100%
17ESE007	90%	90%	70%	35%	75%	35%	85%
17ESE010	85%	35%	65%	10%	75%	35%	85%
17ESE012	75%	30%	40%	10%	65%	25%	85%

B

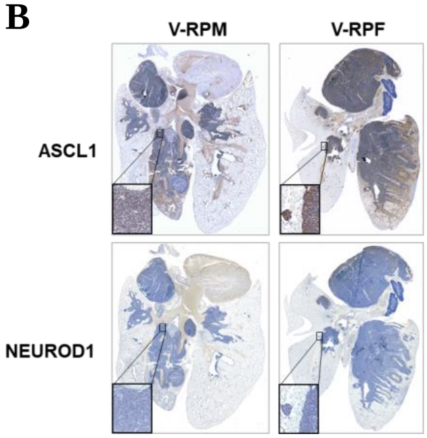

Figure S2. Related to Figure 3.
(A) Estimation of staining intensities of different antibodies on lungs of vehicle- and cisplatin-treated RPM and RPF mice. Staining intensities are averaged across lesion types and therefore do not reflect the expression heterogeneity seen for many of the markers (see Figure S2B). **(B)** Representative IHC stainings of ASCL1 and NEUROD1 in RPM and RPF lungs, respectively. Insets show nuclear staining of ASCL1 in the SCLC cells of the advanced lesions from RPM lung and of initial and advanced lesions from RPF lung, whereas the staining of NEUROD1 was largely absent (scale bar 50 µm).

Figure S3. Related to Figures 4 and 5

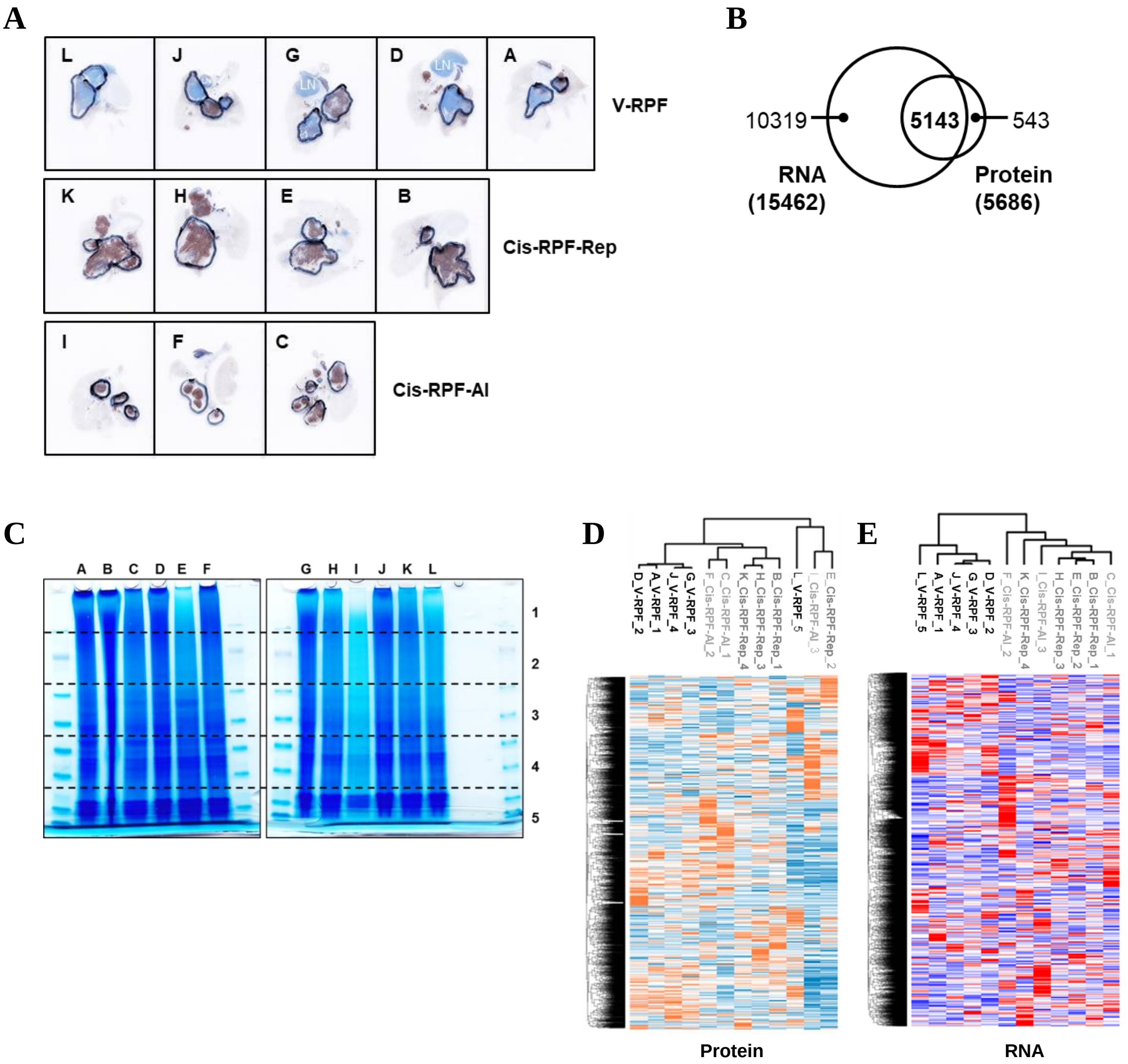

Figure S3. Related to Figures 4 and 5.
(A) CDH1 stainings of vehicle- and cisplatin-treated lung samples used for proteomic and transcriptomic analyses. Of note, all circled tumor areas belonging to one lung were combined, resulting in a total of 5 V-RPF, 4 Cis-RPF-Rep and 3 Cis-RPF-Al samples. LN, lymph node **(B)** Total number of identified genes on RNA and protein level using RNA-Seq and LC-MS/MS analysis, respectively. **(C)** Coomassie-staining and cutting scheme of protein samples separated by SDS-polyacrylamide gel electrophoresis. **(D), (E)** Unsupervised hierarchical clustering of all identified genes using z-score values of normalized spectral counts (LC-MS/MS analysis, (D)) and normalized reads (RNA-Seq analysis, (E)). Relative protein abundancies (D) are shown from low (light blue) to high (orange), relative RNA levels (E) from low (dark blue) to high (red).

Figure S4. Related to Figure 4

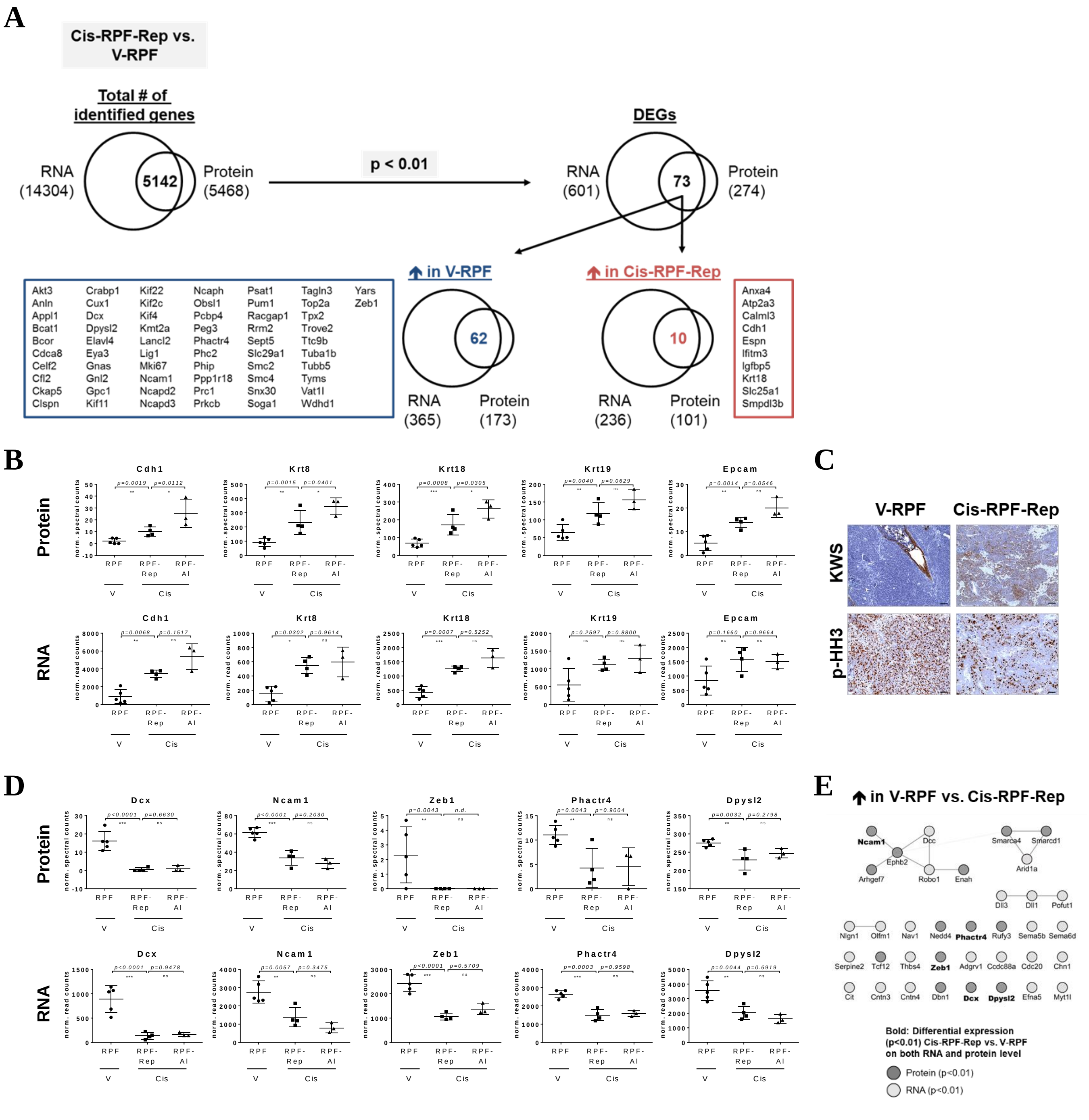

Figure S4. Related to Figure 4.
(A) Overview statistical filtering strategy Cis-RPF-Rep vs. V-RPF comparison and overlap of genes identified in transcriptomic and proteomic analyses (differentially expressed genes determined using beta-binomial test on normalized spectral counts for protein and DESeq2 for RNA-seq data). **(B)** Expression plots of *Cdh1* and functionally connected epithelial marker proteins that were significantly differentially expressed on protein level (p-value * < 0.05, ** < 0.01, *** < 0.001). **(C)** Wide-spectrum keratin (KWS) and phospho-histone H3/p-HH3 (Ser10) stainings of V-RPF and Cis-RPF-Rep samples **(D)** Expression plots of migration-associated genes of neuronal origin that were significantly differentially expressed on both protein and RNA level. (**E**) Migration-associated genes of neuronal origin with highly significant (p < 0.01) decrease in protein and/or RNA levels in Cis-RPF-Rep vs. V-RPF comparison.

Figure S5. Related to Figure 4

A

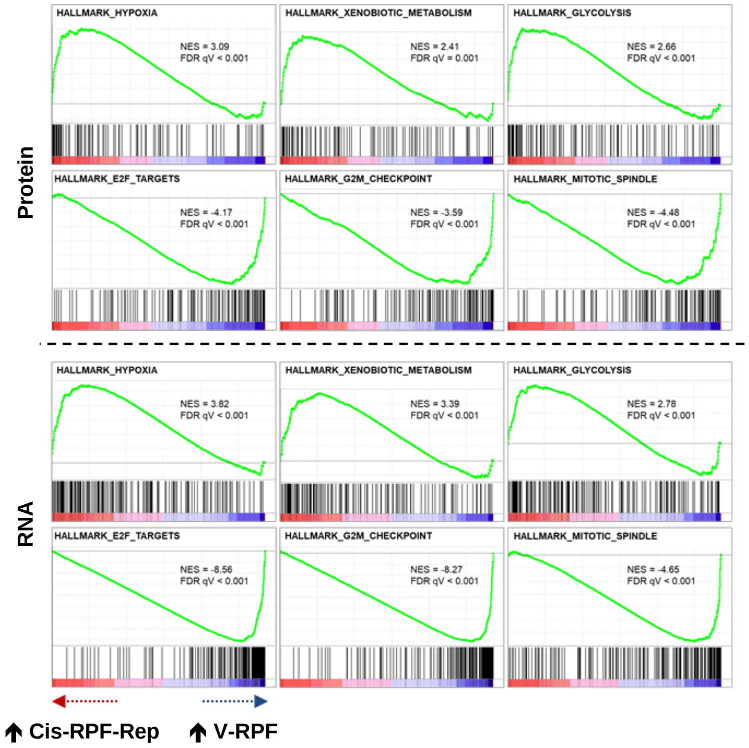

B

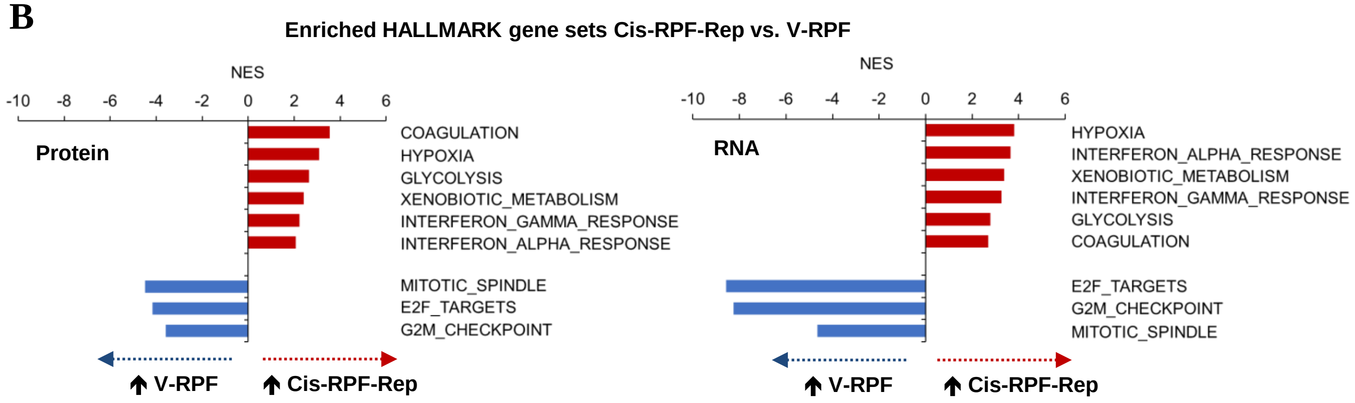

C

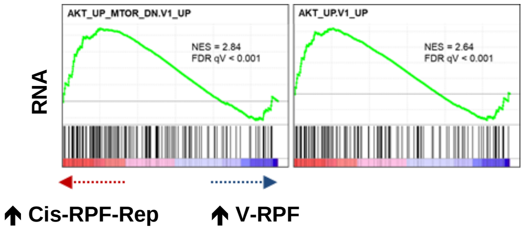

Figure S5. Related to Figure 4.

(A) Enrichment plots of gene sets significantly (FDR q-value<0.05, normalized enrichment score, NES ≥ 2.5) enriched on both protein and RNA level (GSEA_HALLMARKS) in Cis-RPF-Rep vs. V-RPF comparison. **(B)** Gene sets significantly (FDR q-value < 0.05, normalized enrichment score, NES ≥ 2.5) enriched on both protein and RNA level in Cis-RPF-Rep vs. V-RPF comparison (GSEA_HALLMARKS). Figure 4C shows averages (av. NES) of the separate protein and RNA NES values shown here. **(C)** Enrichment plots of oncogenic signature gene sets AKT_UP_MTOR_DN.V1_UP and AKT_UP.V1_UP enriched on RNA level in Cis-RPF-Rep vs. V-RPF comparison.

Figure S6. Related to Figure 5

A

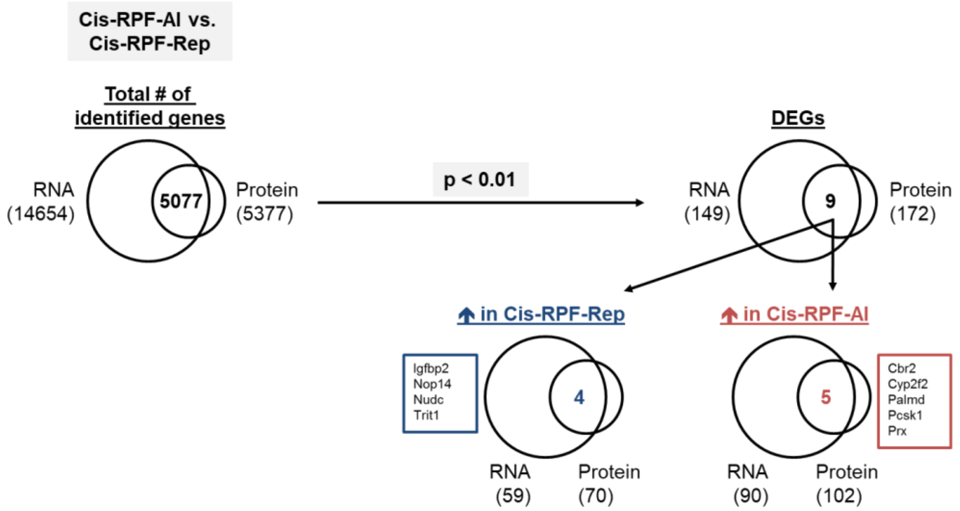

B

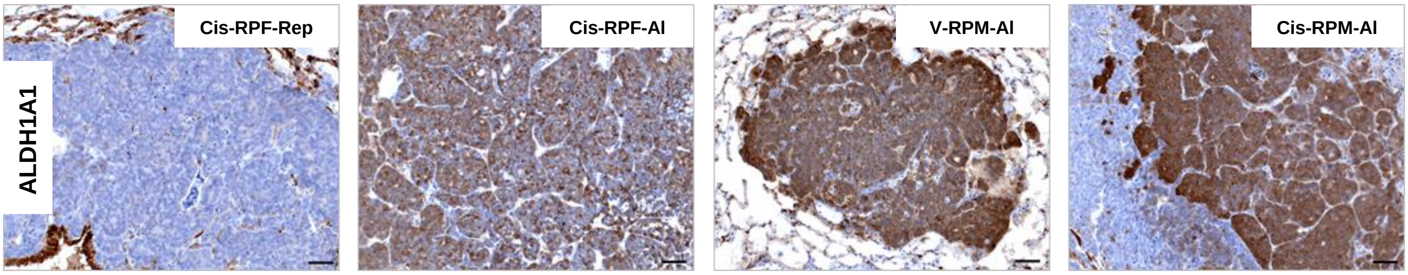

C

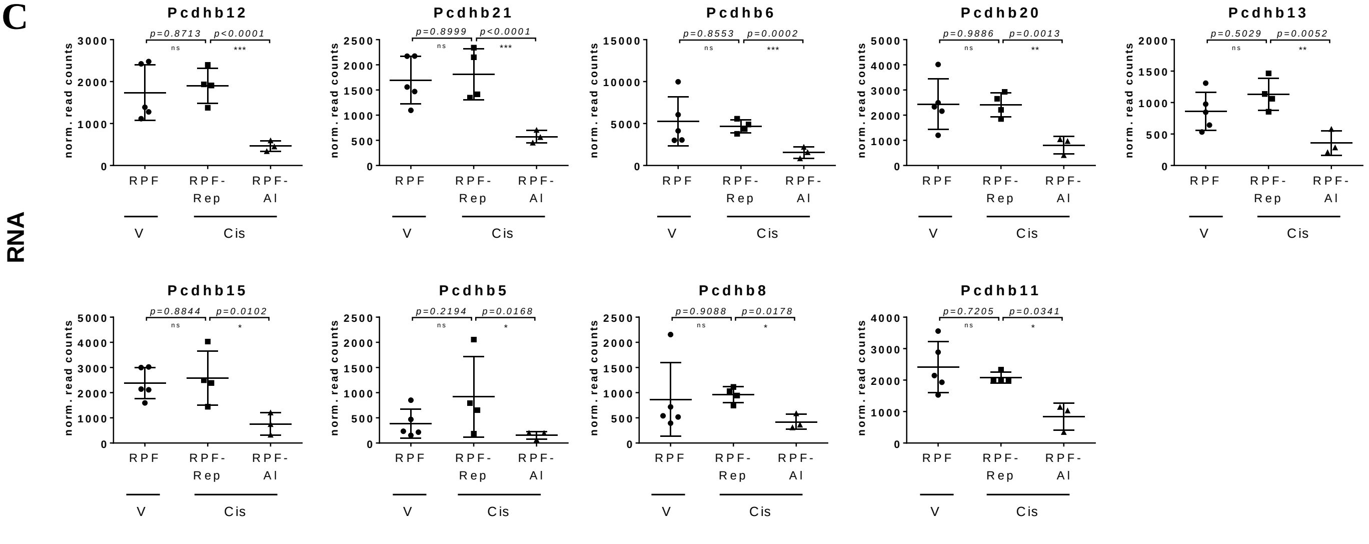

Figure S6. Related to Figure 5.
(A) Overview statistical filtering strategy Cis-RPF-Al vs. Cis-RPF-Rep comparison and overlap of genes identified in transcriptomic and proteomic analyses (differentially expressed genes determined using beta-binomial test on normalized spectral counts for protein and DESeq2 for RNA-seq data). **(B)** ALDH1A1 stainings of repopulating and alveolar populations in RPF and RPM lung samples. **(C)** Expression plots of significantly differentially expressed *Pcdhb* protocadherin genes (p-value * < 0.05, ** < 0.01, *** < 0.001).
